# Transcranial direct current stimulation with Bosu-ball training increases cortical activation and improves ankle-foot function among individuals with chronic ankle instability: A randomized controlled trial

**DOI:** 10.1371/journal.pone.0342751

**Published:** 2026-02-27

**Authors:** Xin Luo, Peixin Shen, Xueke Huang, He Gao, Yubin Ge, Zhiwen Dong, Yan Chen, Daniel T.P. Fong, Qipeng Song

**Affiliations:** 1 Graduate School, Shandong Sport University, Jinan, China; 2 College of Sports and Health, Shandong Sport University, Jinan, China; 3 School of Sport, Exercise and Health Sciences, Loughborough University, Loughborough, United Kingdom; Iran University of Medical Sciences, IRAN, ISLAMIC REPUBLIC OF

## Abstract

**Objectives:**

This study explored the effects of High-definition transcranial direct current stimulation (HD-tDCS) with Bosu-ball training on cortical activation and ankle-foot function among individuals with chronic ankle instability (CAI).

**Design:**

Single-blind randomized sham-controlled trial.

**Setting:**

Biomechanics laboratory.

**Methods:**

Thirty-seven participants were allocated to tDCS+Bosu group (n=19) and Bosu group (n=18), received 6-week active or sham HD-tDCS with Bosu ball training. Change values of oxyhemoglobin concentration (ΔHbO_2_) and Foot and Ankle Ability Measure (FAAM) were measured pre- and post-intervention. Two-way analysis of variance and Pearson correlation analysis were applied.

**Results:**

We detected significant group-by-time interactions in ΔHbO_2_ for the affected and non-affected side premotor cortex and supplementary motor area (PMC & SMA, channel 4: P=0.048; channel 14: P=0.047) and the affected side primary motor cortex (M1, channel 6: P=0.049), and significant time effects for the affected and non-affected side PMC & SMA (channel 2: P=0.043; channel 12: P=0.047), M1 (channel 5: P=0.041; channel 15: P=0.027; channel 16: P=0.049), primary somatosensory cortex (S1, channel 7: P=0.039; channel 17: P=0.043) and somatosensory association cortex (SAC, channel 8: P=0.021; channel 19: P=0.035). We detected significant interaction in the FAAM Sports subscale (P=0.046), and significant time effect in the FAAM Activities of Daily Living subscale (ADL subscale) (P<0.001). The increments in ΔHbO_2_ of channels 4, 5, 16, 19 were moderately positively correlated with the increment in scores of the FAAM Sports subscale (P=0.015, P=0.006, P=0.008, P=0.003). The increment in ΔHbO_2_ of channel 7 was weakly positively correlated with the increment in scores of the FAAM Sports (P=0.049) and ADL subscales (P=0.038).

**Conclusions:**

Bosu-ball training increased cortical activation and improved ankle-foot function among individuals with CAI, and yielded more improvements with active HD-tDCS. The increment of cortical activation was positively correlated with the improvement of ankle-foot function.

## Introduction

Ankle sprains are the most common sports injury, accounting for 10%−30% of all sports-related injuries [1]. In the United States, approximately 2,000,000 ankle sprains occur annually [1], resulting in a cost of about $20 billion [2]. Elite players experience 2–3 days of training loss per ankle sprain [3], and about one-quarter of people with ankle sprains are unable to return to school or work for more than seven days post-injury [4]. Following acute ankle sprains, approximately 40% of people develop chronic ankle instability (CAI), characterized by recurrent sprains and ankle-foot dysfunction [5]. CAI is normally considered a kind of musculoskeletal injury [6], conventional rehabilitation for CAI such as strength training, balance training, etc. primarily focuses on localized symptoms and impaired function, but the risk of recurrent sprains remains high [2].

Deficiencies in cortical activation have been noted among individuals with CAI, potentially contributing to recurrent sprains and ankle-foot dysfunction [7,8]. Recent research suggests that CAI should be considered a neurophysiological dysfunction involving maladaptive neuroplastic changes in the cerebral cortex [8]. Specifically, individuals with CAI exhibit reduced cortical excitability in the primary motor cortex (M1) compared to those without CAI, potentially leading to altered movement patterns [9]. Compared to uninjured individuals, individuals with CAI presented significantly lower preparatory brain activity at the supplementary motor area (SMA), M1, frontal cortex, and primary somatosensory cortex (S1) during gait initiation, which is associated with impaired anticipatory postural adjustments, motor preparation and planning, and proprioception [10]. One study showed that task-related activation of the right paracentral lobule and postcentral gyrus, right supplementary motor area, left precentral and postcentral gyrus was significantly lower among individuals with CAI than that of those without CAI during motor tasks, and the lower activation of left precentral gyrus was positively correlated with poorer plantarflexion proprioception among individuals with CAI [11]. Cortical activation during motor tasks can be assessed by functional near-infrared spectroscopy (fNIRS) [7], a neuroimaging technique based on neurovascular coupling with both favorable spatial and temporal resolution [12]. Compared to electroencephalography (EEG) applied in Beyraghi et al.’s study [10], fNIRS exhibits greater robustness to motion artifacts, and provide better portability compared to functional magnetic resonance imaging (fMRI) in capturing cortical activity in dynamic scenarios [12]. One fNIRS study showed that activation of primary somatosensory cortex (S1) was significantly lower than that of copers (those who have experienced ankle sprains but did not develop CAI) during single-leg stance [13].

Transcranial direct current stimulation (tDCS) may be an effective way in increasing cortical activation and improving ankle-foot function. tDCS is a non-invasive brain stimulation technique, which can change the resting membrane potential and reduce the depolarization threshold of the membrane of neurons by applying low current on the scalp, and thus increase cortical excitability [14]. Studies have demonstrated that tDCS is effective in enhancing cortical activation of the somatosensory cortex [15] and motor cortex [16], and improving ankle-foot sensorimotor function [17]. Up to now, a total of eleven longitudinal studies have explored the effectiveness of tDCS on individuals with CAI [9,17–26], while only three studies included cortical activity characteristics among this population [9,23,24]. However, inconsistent findings exist in these studies. Bruce et al. demonstrated that corticospinal excitability of lower limb muscles can be enhanced by active tDCS [9]. In contrast, Needle et al. didn’t report significant improvements of active tDCS or sham tDCS on corticospinal and segmental excitability [23], and Beyraghi & Khanmohammadi found no additive effect of active tDCS on preparatory brain activities among individuals with CAI [24]. Previous studies applied conventional tDCS to explore its effectiveness on cortical activities among individuals with CAI [9,23,24]. Compared to conventional tDCS, High-definition tDCS (HD-tDCS) can be seen as an optimized stimulation protocol [27]. The HD-tDCS adopts 4 × 1 ring electrodes arrangement, which can restrict the area of electrical stimulation to the range of electrode arrangement, localize the peak of electric field at the central electrode [28], reduce the stimulation to non-target areas [29], and thus provide effective targeted cortical stimulation with favorable penetrability [28].

The combination of HD-tDCS and Bosu-ball training may further increase cortical activation and improve ankle-foot function among individuals with CAI. Studies showed that active tDCS with physical training can increase cortical excitability [30] and improve ankle-foot function more significantly than sham tDCS with physical training among this population [9,17]. The previous three tDCS-CAI studies applied distinct physical training to pair with tDCS. Bruce et al. adopted evertor strength training, which mainly focus on muscle strength [9]; Beyraghi & Khanmohammadi applied a balance training program which included single-leg stance, single-leg hopping to stabilization and hop-to-stabilization and reach tasks [24], and Needle et al applied a motor planning training program which included obstacle walking, dual-task balance, and agility exercises [23]. Compared to these two training programs, Bosu-ball training program is more capable of simulating the scenario when an ankle sprain occurs. Bosu-ball training is a balance training method, which allows trainees to counteract continuous perturbations to the ankle and center-of-gravity through movements like maintaining single-leg balance on the unstable hemispherical surface of the Bosu-ball [26], thereby promoting the restoration of impaired ankle-foot sensorimotor function [31,32]. Research team has demonstrated that synchronous active HD-tDCS with Bosu-ball training can decrease ankle injury potential during drop-landing [26] and side-cutting tests [20], and improve postural stability more significantly than sham HD-tDCS with Bosu-ball training among individuals with CAI [19], albeit effects of the intervention on cortical activation has not been demonstrated by any other studies yet.

Deficiencies in cortical activation could lead to ankle-foot dysfunction and recurrent sprains among individuals with CAI, and HD-tDCS with Bosu-ball training holds potential to increase cortical activation and improve ankle-foot function. This study aims to explore the effects of HD-tDCS with Bosu-ball training on cortical activation and ankle-foot function among individuals with CAI. Our hypotheses are: (1) Active HD-tDCS with Bosu-ball training can increase cortical activation of the premotor cortex and supplementary motor area (PMC & SMA), M1, S1 and somatosensory association cortex (SAC) more significantly than sham HD-tDCS with Bosu-ball training among individuals with CAI, reflected by higher change values of oxyhemoglobin concentration (ΔHbO_2_) during single-leg stance; (2) Active HD-tDCS with Bosu-ball training can improve ankle-foot function more significantly than sham HD-tDCS with Bosu-ball training among individuals with CAI, reflected by higher scores of Foot and Ankle Ability Measure (FAAM) Sports and Activities of Daily Living (ADL) subscales; and (3) The increment of ΔHbO_2_ in measured cortical areas is positively correlated with the increment of scores of FAAM Sports and ADL subscales, reflected by the Pearson correlation coefficient (r).

## Materials and methods

### Ethical approval

All participants signed the approved written informed consent forms before participation in this study. This study was approved by the Ethics Committee of Shandong Sport University (2023035) and was in accordance with the Declaration of Helsinki. The individual in this manuscript has given written informed consent (as outlined in PLOS consent form) to publish these case details.

### Protocol registration

The authors confirm that all ongoing and related trials for this drug/intervention are registered. This trial has been registered post-recruitment in the Chinese Clinical Trial Registry (ChiCTR) due to an unexpected oversight by the personnel specifically responsible for clinical trial registration, registration number: ChiCTR2500098875, registration date: 2025.03.14. URL: https://www.chictr.org.cn.

### Study design

This is a single-blinded, randomized, sham-controlled, superiority study with a parallel group design, complying with CONSORT reporting guidelines [33]. Thirty-seven participants were assigned to the tDCS+Bosu group or the Bosu group based on the random numbers generated by Excel 2021 software (Microsoft Office, Redmond, Washington, USA) at an approximate ratio of 1:1, with 19 in the tDCS+Bosu group and 18 in the Bosu group. Details of the assigned group were written on cards and concealed using sequentially numbered opaque sealed envelopes. The allocation sequence and participants' assignment were conducted by independent personnel. The personnel who enrolled and assigned participants to the interventions had no access to the allocation sequence. Participants in the Bosu group received sham HD-tDCS with Bosu-ball training, and those in the tDCS+Bosu group received active HD-tDCS with Bosu-ball training for a total of 6 weeks with three 20-min sessions per week. Participants were blinded after assignment to interventions, and were excluded for their absence or reception of any other treatments or training. All interventions and comparators were delivered as intended. Cortical activation of the PMC & SMA, M1, S1 and SAC, and the scores of FAAM Sports and ADL subscales were measured before (week_0_) and after (week_7_) the intervention.

### Participants

An a priori power analysis conducted using the G*Power 3.1 software (University of Düsseldorf, Düsseldorf, Germany) indicated that a minimum of 26 participants (total sample for two groups) should be recruited to obtain an alpha level of 0.05 and a statistical power of 0.90 based on a previous study: From pre- to post-intervention (an 18-day tDCS intervention), the ΔHbO_2_ decreased in the right dorsolateral prefrontal cortex (DLPFC) during the Color-Word Stroop test among the healthy undergraduates who received a total of 9 sessions of active tDCS intervention while increased among the healthy undergraduates who received a total of 9 sessions of sham tDCS intervention, with a significant group-by-time interaction (P = 0.040, η^2^_p_=0.100, Cohen’s *d* = 0.417 for the ΔHbO_2_ of right DLPFC in Anodal group from pre- to post intervention, and Cohen’s *d* = −0.359 for the ΔHbO_2_ of right DLPFC in Sham group from pre- to post intervention) detected in the ΔHbO_2_ using a two-way analysis of variance (ANOVA) with repeated measures [34]. A previous study reported an attrition rate of approximately 25% in a six-week exercise intervention study [35]. Therefore, a minimum of 33 participants needed to be successfully enrolled and randomized to ensure the final analyzable sample size met the requirement.

We screened 75 people from Shandong Sport University through posters and distributing fliers from August 1, 2024 to September 1, 2024 for eligibility. The participants who were recruited earlier took part in the subsequent recruitment of other participants. The study lasted approximately three months for all the tests and intervention procedures in the biomechanics laboratory of Shandong Sport University. The pretest was conducted in September, and the posttest in November 2024. All tests and intervention procedures were conducted by qualified therapists, who had received strict training prior to the initiation of the tests and interventions.

**Fig 1:**
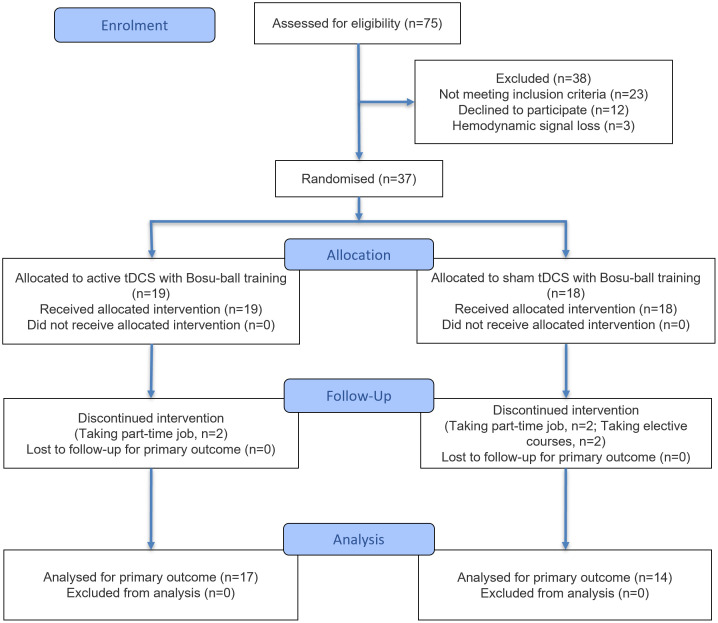
The CONSORT diagram for flow of participants. Data from 31 participants were analyzed eventually. A total of 44 participants were excluded from the eligibility assessment. tDCS, transcranial direct current stimulation.

The CONSORT diagram for flow of participants was shown in Fig 1. The inclusion criteria adhered to the guidelines established by the International Ankle Consortium [36]. We required eligible participants to have experienced at least one severe ankle sprain one year prior, resulting in pain, swelling, and other inflammatory symptoms that hindered participation in daily activities for more than one day; to be aged 18–24 years, regardless of gender; to have experienced at least two episodes of ankle “giving way” within the past six months; to have a persistent feeling of ankle instability and functional impairment during daily activities; and to have a score < 24 of the Cumberland Ankle Instability Tool (CAIT). The exclusion criteria included a history of lower-extremity fracture or surgery within the past year, acute injuries such as lower-extremity sprains within the past three months, bilateral CAI, electrical stimulation intolerance, and neurological disorders that affect motor function.

### Bosu-ball training protocol

**Fig 2:**
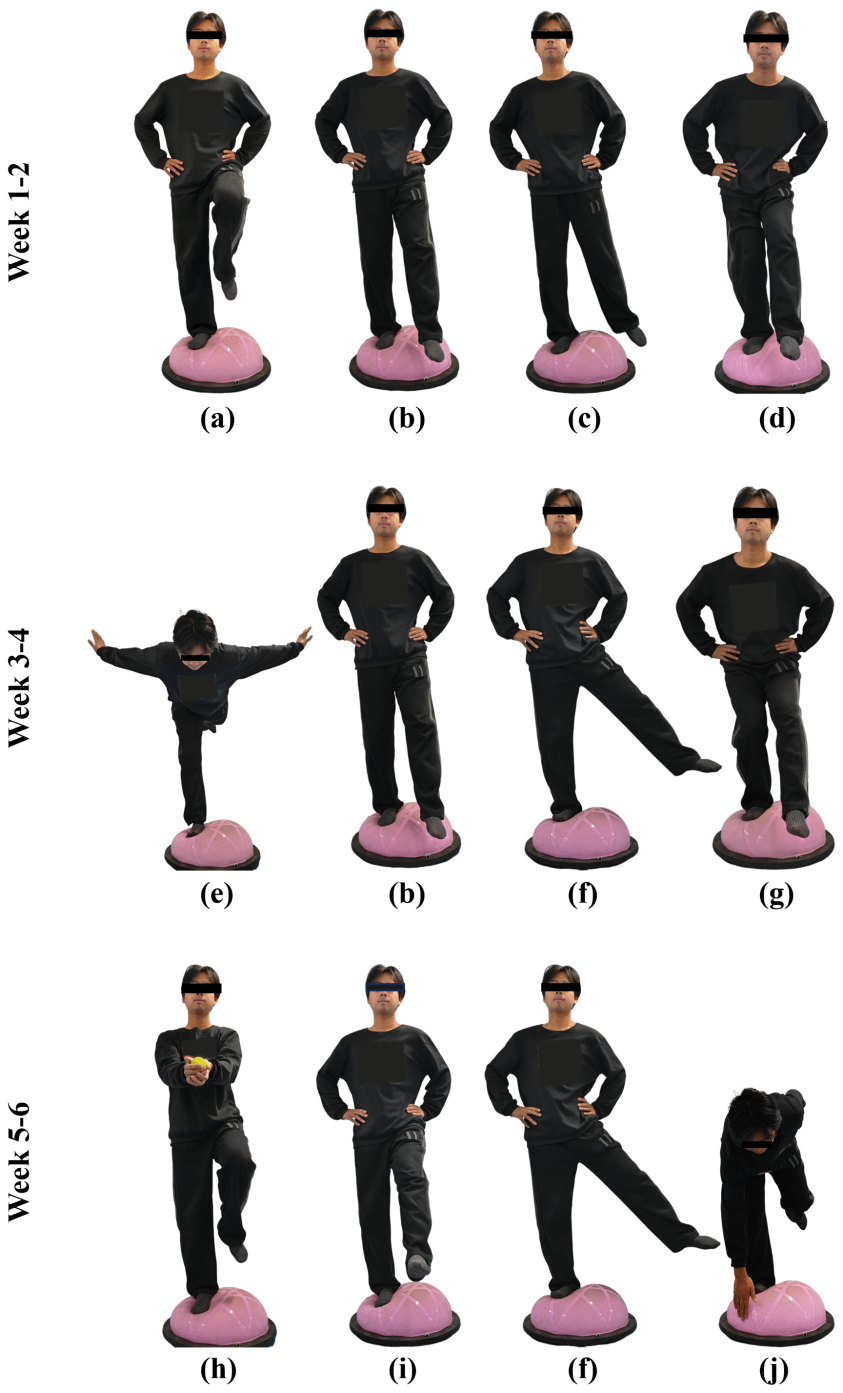
6-week Bosu-ball training program. (a) Single-leg stance; (b) Single-leg stance with leg swing forward-backward (30°-45°); (c) Single-leg stance with leg swing medially-laterally (20°-30°); (d) Single-leg stance with knee bend (20°-30°); (e) Swallow balance; (f) Single-leg stance with leg swing medially-laterally (30°-45°); (g) Single-leg squat; (h) Tossing and catching a tennis ball while maintaining single-leg stance; (i) Single-leg stance with leg swing forward-backward (45°-60°); (j) Bending over to touch the edge of the Bosu-ball while maintaining single-leg stance.

Participants stood on their affected leg with shoes taken off on the lateral side of the Bosu-ball (58 cm in diameter, SPXine, Shandong, China). The protocol consisted of a total of 10 movements, including (a) Single-leg stance; (b) Single-leg stance with leg swing forward-backward (30°–45°); (c) Single-leg stance with leg swing medially-laterally (20°–30°); (d) Single-leg stance with knee bend (20°–30°); (e) Swallow balance; (f) Single-leg stance with leg swing medially-laterally (30°–45°); (g) Single-leg squat; (h) Tossing and catching a tennis ball while maintaining single-leg stance; (i) Single-leg stance with leg swing forward-backward (45°–60°); (j) Bending over to touch the edge of the Bosu-ball while maintaining single-leg stance. Each movement was performed for 30s and repeated 5 times, with a 30s rest between movements for a total of approximately 20 min each session; the protocol adopted a combination of different movements in a progressive manner for a total of 6 weeks (Fig 2), synchronized with HD-tDCS intervention.

### HD-tDCS intervention protocol

**Fig 3:**
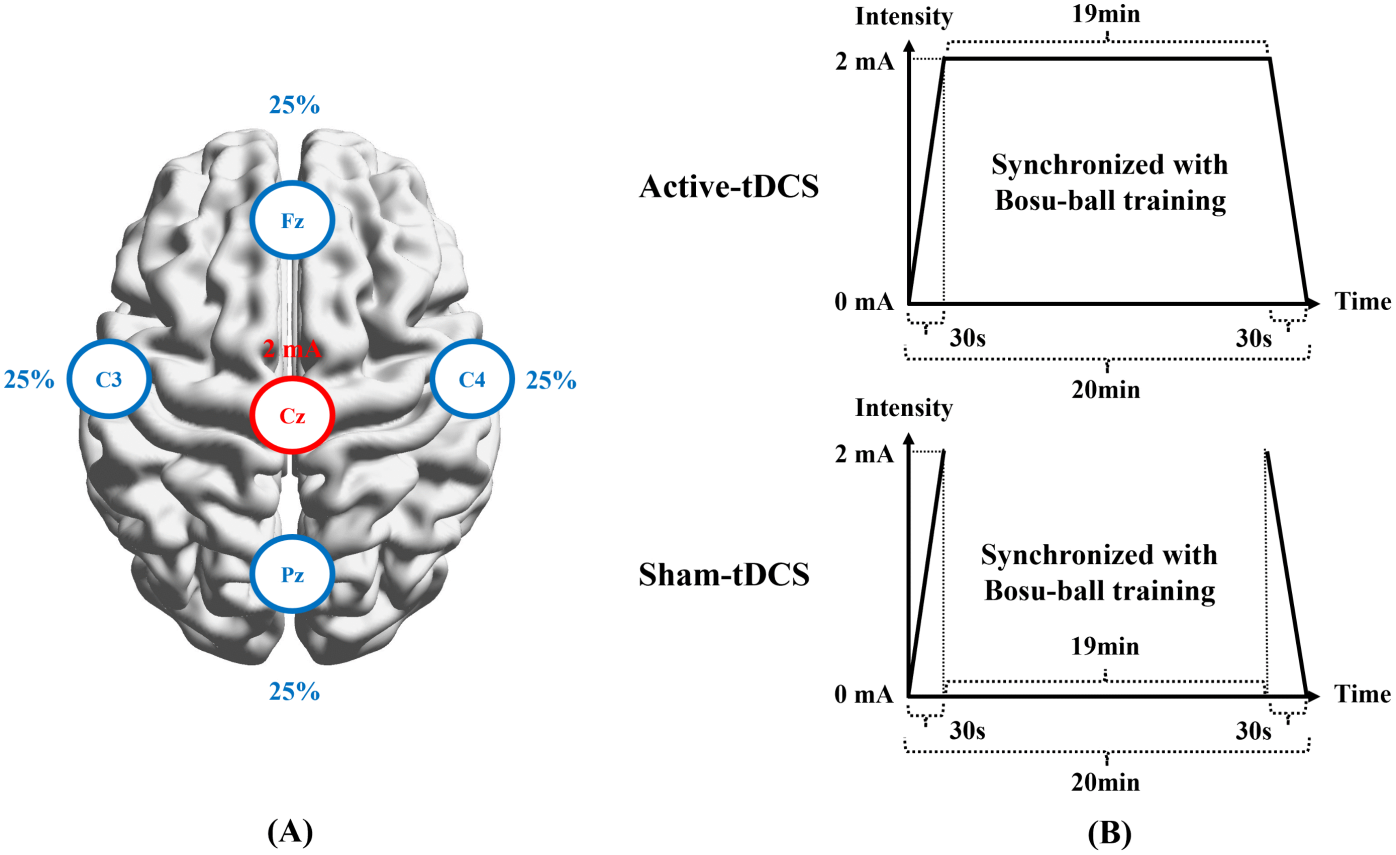
(A) Electrode arrangement and (B) time-intensity settings of HD-tDCS. tDCS, transcranial direct current stimulation.

A tDCS device (Starstim8, Neuroelectronics, Spain) was used to deliver stimulation. Five sponge electrodes (8 cm²) soaked in 0.9% saline were placed in a neoprene cap according to the international 10/20 Electroencephalogram (EEG) system and a 4 × 1 ring HD-tDCS electrode montage [17]. The anode was at Cz, and the other electrodes were at Fz, Pz, C3, and C4. The current intensity at the anode was set to 2 mA, with a 100% current return ratio evenly distributed across the remaining four electrodes (Fig 3A) [26]. The current ramped up from 0 mA to 2 mA in 30s, maintained at 2 mA for 19 min, and ramped down to 0 mA in the last 30s, totaling 20 min stimulation synchronized with Bosu-ball training (Fig 3B) [26]. For sham tDCS, the current ramped up from 0 mA to 2 mA in the first 30s, and ramped down from 2 mA to 0 mA in the last 30s, while no current was delivered during the middle 19 min (Fig 3B).

### fNIRS test protocol

Hemodynamic data during single-leg stance were collected using a portable fNIRS device (Lightnirs, Shimadzu Corp., Kyoto, Japan) with 8 emitters and 8 detectors, forming 20 channels. The optode configuration covered PMC & SMA, M1, S1 and SAC [7], with a 3 cm distance between adjacent optodes. Each optode had 3 diodes with wavelengths of 780 nm, 805 nm, and 830 nm at a sampling rate of 13.3 Hz [7]. A 3-D digitizer (FASTRAK, Polhemus, Vermont, USA) was used to conduct spatial registration to determine Montreal Neurological Institute (MNI) coordinates of each channel and corresponding brain areas based on the NIRS-SPM toolkit [37], as presented in S1 Table. Channels 1–4 mapped to the left PMC & SMA; channels 11–14 to the right PMC & SMA; channels 5–6 to the left M1; channels 15–16 to the right M1; channel 7 to the left S1; channel 17 to the right S1; channels 8–10 to the left SAC; and channels 18–20 to the right SAC.

Before the test, participants practiced one trial (60s rest + 30s single-leg stance) for familiarization. They then sat quietly for 60s to measure resting cortical activity. Next, they stood with arms on their waists and eyes on a wall marker for 10s to minimize head movements and mind wandering, and eliminate hemodynamic changes induced by sit-to-stand transfer movements. Upon hearing “start”, they performed single-leg stance on their affected leg while lifting the other leg with the hip flexed to 30° and the knee flexed to 45° for 30s [7]. This procedure was repeated three times, with 60s of rest between each trial.

### Adverse events monitoring

Throughout the 6-week intervention (3 sessions/week) and pre-/post-intervention assessments, adverse events were monitored systematically. Two types of potential adverse events were focused on: (1) HD-tDCS-related events (e.g., scalp skin reactions, dizziness, nausea, etc.) and (2) Bosu-ball training-related events (e.g., ankle injury, muscle soreness, etc.).

Before and after each intervention session, trained therapists conducted face-to-face inquiries (e.g., “Have you experienced scalp tingling/itching, dizziness, or other discomfort?”) and visual observations (e.g., scalp skin condition, gait abnormalities) to collect adverse event information.

An Adverse Event Record Form (S2 Table, based on the FDA MedWatch Form 3500, and previous studies reported the adverse events of tDCS intervention [38,39] and CAI exercise intervention [40,41]) was established for each participant to document the occurrence time, type, severity, duration, and management of any adverse events in detail.

### Outcomes

There were two primary outcomes in this study. The first one was the ΔHbO_2_ in PMC & SMA, M1, S1 and SAC. The Homer 2 toolbox based on MATLAB (R2013b, MathWorks Inc., Natick, United States) was used to analyze hemodynamic data [42]. The raw optical intensity data were converted to optical density and then corrected for motion artifacts using Temporal Derivative Distribution Repair algorithms [43]. Band-pass filtering (0.01–0.1 Hz) removed instrumental noise, baseline drift, and physiological noise [44]. The modified Beer-Lambert law converted optical density to HbO_2_ concentration [45]. Baseline correction was done using the last 5s of each sitting period’s mean value of HbO_2_ concentration to acquire the change values of HbO_2_ concentration (ΔHbO_2_) during single-leg stance relative to the sitting period. Data from three valid single-leg stance tasks were superimposed and averaged for the subsequent statistical analysis. To facilitate the interpretation of results, the hemodynamic data of bilateral cerebral hemispheres from participants with left-sided CAI were subjected to axisymmetric matching. Therefore, the hemodynamic data from the left cerebral hemisphere (Channels 1–10) represent the cortical areas primarily innervating the affected lower limb, which is labeled as the “affected side” cortical areas, while those from the right cerebral hemisphere (Channels 11–20) represent the cortical areas primarily innervating the unaffected lower limb, which is labeled as the “non-affected side” cortical areas. Cortical activation maps were visualized using the BrainNet Viewer toolkit [46].

Another primary outcome was the scores of FAAM Sports and ADL subscales. Participants completed the scales based on their actual conditions over the past week. The FAAM consists of a 21-item ADL subscale and an 8-item Sports subscale, with each item scored from 0 to 4. Based on the maximum scores of each subscale (84 for the 21-item ADL subscale and 32 for the 8-item Sports subscale), obtained scores were converted to percentage scores. A higher percentage score indicates better ankle-foot function [47]. Results were retained to two decimal places.

The secondary outcome of this study was the correlation between the increment of ΔHbO_2_ in measured cortical areas and the increment of scores of FAAM Sports and ADL subscales, as reflected by the Pearson correlation coefficient (r).

### Statistical analysis

We performed statistical analysis using SPSS 21.0 software (IBM SPSS, Armonk, NY, USA). All data are presented as mean ± standard deviation (M ± SD). The normality of the data was verified using Shapiro-Wilk tests. A two-way ANOVA with repeated measures was employed to examine the main effects of group (tDCS+Bosu group vs. Bosu group) and time (week_0_ vs. week_7_), as well as the group-by-time interaction. In the event of a significant interaction, Bonferroni-adjusted simple effects analysis was utilized to conduct pairwise comparisons. Partial eta square (η^2^_p_) was used to represent the effect size of interaction effects. The thresholds for η^2^_p_ were as follows: 0.01–0.06, small; 0.06–0.14, moderate; > 0.14, large. A 95% confidence interval (95% CI) was used to represent the effect size of time main effect, group main effect and the simple effects analysis. Pearson correlation analysis (for normally distributed data) or Spearman’s rank correlation coefficient (for non-normally distributed data) was used to verify the correlation between the increment of ΔHbO_2_ in measured cortical areas and the increment of scores of FAAM Sports and ADL subscales, regardless of groups. Channels with significant statistical differences identified by two-way ANOVA with repeated measures were included. The thresholds for Pearson correlation coefficient (r) and Spearman’s rank correlation coefficient (ρ) were as follows: 0–0.4, weak; 0.4–0.7, moderate; 0.7–1.0, strong, with the sign indicating the direction of correlation (positive for a positive correlation and negative for a negative correlation).

## Results

### Baseline characteristics

The Shapiro-Wilk test showed that all data except that used for correlation analysis were normally distributed*.* After the intervention, 17 participants remained in the tDCS+Bosu group (age: 20.5 ± 0.9 years; height: 175.3 ± 8.3 cm; body mass: 71.1 ± 8.4 kg; CAIT score: 15.6 ± 5.3) and 14 in the Bosu group (age: 21.2 ± 1.7 years; height: 173.2 ± 11.9 cm; body mass: 68.9 ± 11.6 kg; CAIT score: 16.0 ± 3.9), respectively. Data from the 31 participants were included in the final analysis, while the data of excluded participants were not. There were no significant statistical differences in age, height, body mass and CAIT scores between the two groups detected by independent samples t-test (P > 0.05) (S3 Table).

None of the data from 7 attrited participants were included in the final statistical analysis. For the 31 participants who completed the entire intervention and pre-/post-intervention assessments, there were no missing values in outcome variables (ΔHbO₂ and FAAM scores) or baseline characteristics. The dataset used for final statistical analysis was complete with no missing entries.

### Adverse events

Severe adverse events: No severe adverse events (e.g., severe dizziness/vomiting, severe skin redness/ulceration, recurrent ankle sprains requiring medical intervention) were reported in either the tDCS+Bosu group or the Bosu group during the entire study period.

Mild adverse events: In the tDCS+Bosu group, 2 participants reported mild scalp tingling during the early stage of intervention (first 2 sessions). The symptom had a duration of < 5 min and disappeared with adaptation, requiring no special treatment. In the Bosu group, 1 participant experienced mild calf muscle soreness, which occurred within 24 hours after intervention and disappeared spontaneously after 48 hours without affecting subsequent interventions. None of these mild adverse events led to participant dropout from the study.

### Hemodynamic data

**Fig 4:**
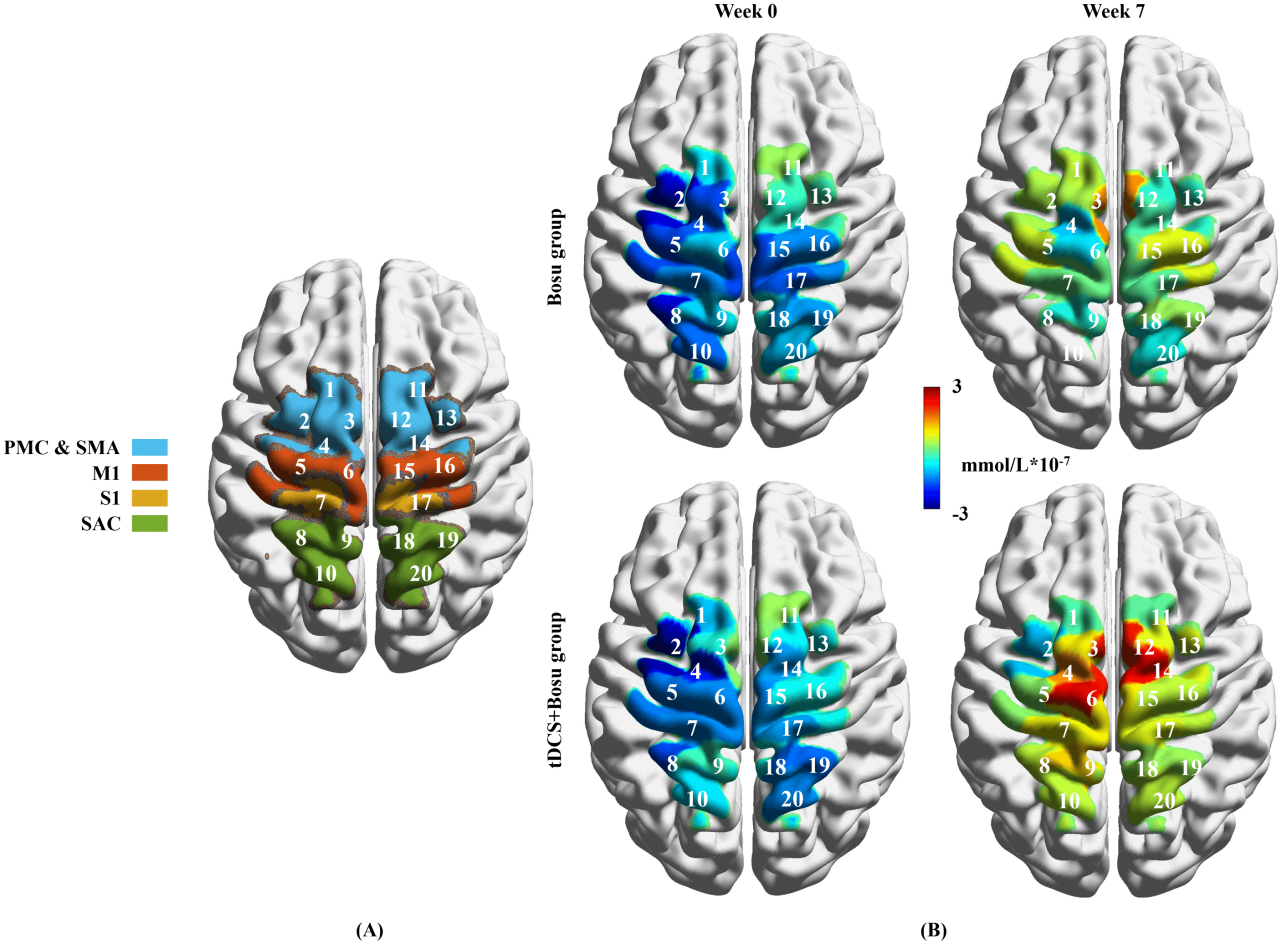
fNIRS channel distribution (A) and ΔHbO_2_ values in each channel by group and time (B). tDCS, transcranial direct current stimulation; PMC & SMA, premotor cortex and supplementary motor area; M1, primary motor cortex; S1, primary somatosensory cortex; SAC, somatosensory association cortex. Channels 1–4 indicated left PMC & SMA; channels 11–14 indicated right PMC & SMA; channels 5–6 indicated left M1; channels 15–16 indicated right M1; channel 7 indicated left S1; channel 17 indicated right S1; channels 8–10 indicated left SAC; and channels 18–20 indicated right SAC.

Fig 4 and Table 1 show the ΔHbO_2_ in measured cortical areas of both groups in week_0_ and week_7_. We detected significant group-by-time interactions in ΔHbO_2_ for both the affected side and non-affected side PMC & SMA (affected side: channel 4: P = 0.048, η²_p_ = 0.128; non-affected side: channel 14: P = 0.047, η²_p_ = 0.129) and affected side M1 (channel 6: P = 0.049, η²_p_ = 0.127). Simple effect analysis showed that the ΔHbO_2_ for both the affected side and non-affected side PMC & SMA increased in the tDCS+Bosu group (channel 4: P = 0.001, 95% CI=[−6.030, −0.975]; channel 14: P = 0.004, 95% CI=[−5.589, −1.317]), while in the Bosu group the changes were not statistically significant from week_0_ to week_7_ (channel 4: P = 0.584; channel 14: P = 0.871). Simple effect analysis showed that the ΔHbO_2_ for affected side M1 (channel 6) increased in the tDCS+Bosu group (P = 0.004, 95% CI=[−6.399, −1.259]), while in the Bosu group the changes were not statistically significant from week_0_ to week_7_ (P = 0.936). We also detected significant time effects in ΔHbO_2_ for both the affected side and non-affected side PMC & SMA (affected side: channel 2: P = 0.043, 95% CI=[−4.250, −0.056]; non-affected side: channel 12: P = 0.047, 95% CI=[−3.424, −0.087]), M1 (affected side: channel 5: P = 0.041, 95% CI=[−3.760, −0.089]; non-affected side: channel 15: P = 0.027, 95% CI=[−3.562, −0.286]; channel 16: P = 0.049, 95% CI=[−2.991, 0.003]), S1 (affected side: channel 7: P = 0.039, 95% CI=[−3.335, −0.166]; non-affected side: channel 17: P = 0.043, 95% CI=[−3.286, −0.110]) and SAC (affected side: channel 8: P = 0.021, 95% CI=[−4.149, −0.403]; non-affected side: channel 19: P = 0.035, 95% CI=[−3.099, −0.169]). The ΔHbO_2_ in these cortical areas increased in both groups from week_0_ to week_7_.

**Table 1:** The ΔHbO_2_ (mmol/L^*^10^−7^) in each channel and brain area in the two groups before and after the intervention (M ± SD).

Brain areas	Ch	Hemisphere	Week	Bosu (n = 14)	tDCS+Bosu (n = 17)	Interaction			Time			Group		
						F	P	η^2^_p_	F	P	95% CI	F	P	95% CI
Premotor cortex and supple-mentary motor area	1	Affected side	0	−0.86 ± 3.34	−1.13 ± 3.81	0.064	0.803	0.002	1.984	0.170	−2.497, 0.455	0.295	0.591	−1.115, 2.015
			7	0.36 ± 2.55	−0.27 ± 2.31									
	2		0	−2.49 ± 4.05	−2.70 ± 5.15	0.285	0.597	0.010	4.467	0.043*	−4.250, −0.056	0.408	0.528	−1.462, 3.007
			7	0.28 ± 3.02	−1.05 ± 4.88									
	3		0	−0.25 ± 4.64	−0.60 ± 4.25	0.284	0.598	0.010	1.243	0.274	−2.750, 0.729	0.011	0.918	−2.042, 1.821
			7	0.25 ± 3.30	0.82 ± 2.85									
	4		0	−1.70 ± 2.90	−2.10 ± 4.75	4.249	0.048*	0.128	8.299	0.007	−3.699, −0.666	1.433	0.241	−2.707, 0.577
			7	−1.12 ± 1.60	1.41 ± 2.12^a^									
	11	Non-affected side	0	0.15 ± 3.44	0.21 ± 4.07	0.023	0.880	0.001	0.075	0.786	−1.587, 2.123	0.006	0.937	−1.774, 1.927
			7	0.03 ± 3.69	−0.18 ± 3.45									
	12		0	0.01 ± 3.75	0.11 ± 4.43	0.131	0.720	0.005	4.293	0.047*	−3.424, −0.087	0.180	0.674	−2.215, 1.411
			7	1.44 ± 2.92	2.14 ± 2.51									
	13		0	−0.38 ± 4.34	−0.77 ± 4.83	0.420	0.522	0.014	0.341	0.564	−2.849, 1.464	0.090	0.766	−2.387, 1.771
			7	−0.45 ± 3.71	0.55 ± 3.38									
	14		0	−0.47 ± 3.02	−1.26 ± 3.55	4.314	0.047*	0.129	5.400	0.027	−3.662, −0.300	1.059	0.312	−2.562, 0.868
			7	−0.28 ± 3.16	2.20 ± 2.81^a^									
Primary motor cortex	5	Affected side	0	−1.79 ± 2.87	−1.47 ± 3.80	0.147	0.704	0.005	4.571	0.041*	−3.760, −0.089	0.001	0.974	−1.917, 1.983
			7	0.52 ± 3.73	0.14 ± 4.31									
	6		0	−1.02 ± 4.12	−1.39 ± 4.04	4.212	0.049*	0.127	4.721	0.038	−4.087, −0.211	1.757	0.195	−3.627, 0.649
			7	−0.91 ± 2.81	2.44 ± 4.63^a^									
	15	Non-affected side	0	−1.75 ± 3.60	−1.40 ± 3.13	0.101	0.753	0.003	5.389	0.027*	−3.562, −0.286	0.530	0.473	−2.310, 1.094
			7	−0.11 ± 3.75	0.76 ± 2.51									
	16		0	−1.35 ± 1.97	−0.74 ± 3.32	0.262	0.612	0.009	4.214	0.049*	−2.991, 0.003	0.062	0.805	−1.931, 1.469
			7	0.56 ± 4.06	0.41 ± 3.42									
Primary somato- sensory cortex	7	Affected side	0	−1.42 ± 2.23	−1.41 ± 5.24	0.299	0.589	0.010	4.695	0.039*	−3.335, −0.166	0.162	0.690	−2.277, 1.398
			7	−0.14 ± 2.73	0.73 ± 3.60									
	17	Non-affected side	0	−1.67 ± 3.33	−1.31 ± 2.42	0.083	0.775	0.003	4.461	0.043*	−3.286, −0.110	0.388	0.538	−2.363, 1.184
			7	−0.23 ± 3.65	0.59 ± 4.08									
Somato-sensory association cortex	8	Affected side	0	−2.35 ± 3.61	−1.81 ± 5.17	0.009	0.925	<0.001	5.943	0.021*	−4.149, −0.403	0.156	0.695	−2.559, 1.673
			7	0.03 ± 3.17	0.38 ± 3.74									
	9		0	−1.04 ± 2.74	−0.44 ± 4.39	0.228	0.637	0.008	0.958	0.336	−2.896, 0.937	1.206	0.281	−2.946, 0.850
			7	−0.56 ± 3.92	0.95 ± 3.58									
	10		0	−1.55 ± 3.27	−0.87 ± 4.24	0.021	0.885	0.001	2.098	0.158	−3.423, 0.563	0.307	0.584	−2.524, 1.437
			7	0.04 ± 3.90	0.43 ± 3.92									
	18	Non-affected side	0	−0.68 ± 3.14	−0.69 ± 4.35	0.112	0.740	0.004	0.560	0.460	−2.373, 1.042	0.071	0.792	−2.166, 1.609
			7	−0.33 ± 3.68	0.24 ± 3.58									
	19		0	−1.25 ± 2.70	−1.61 ± 3.16	0.038	0.848	0.001	4.893	0.035*	−3.099, −0.169	0.071	0.838	−1.593, 2.015
			7	0.23 ± 3.94	0.16 ± 3.97									
	20		0	−1.14 ± 3.58	−1.43 ± 3.67	0.843	0.366	0.028	2.060	0.162	−2.742, 0.389	0.144	0.707	−2.321, 1.485
			7	−0.74 ± 4.08	0.39 ± 3.59									

* Indicates significant statistical differences (P < 0.05); ^a^ Indicates significant statistical differences between week_0_ and week_7_ (P < 0.05).

tDCS, transcranial direct current stimulation; M, mean values; SD, standard deviation; ΔHbO_2_, change values of oxyhemoglobin concentration; Ch, channel; 95% CI, 95% confidence interval.

### Self-reported function data

**Fig 5:**
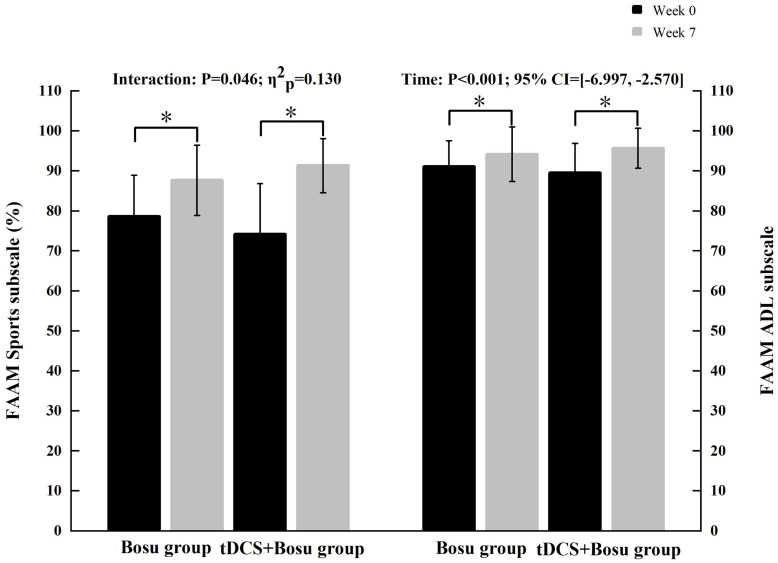
The scores of each FAAM subscale in the two groups before and after the intervention. tDCS, transcranial direct current stimulation; FAAM, Foot and Ankle Ability Measure; ADL, activities of daily living; 95% CI, 95% confidence interval.

Fig 5 and S4 Table show the scores of FAAM Sports and ADL subscales for both groups in week_0_ and week_7_. We detected a significant group-by-time interaction in the Sports subscale (P = 0.046, η²_p_ = 0.130). Simple effect analysis showed that the scores of the Sports subscale increased in both groups, while the increment was greater in the tDCS+Bosu group (P < 0.001, 95% CI=[−22.754, −11.593]) compared to the Bosu group (P = 0.004, 95% CI=[−15.272, −2.836]) from week_0_ to week_7_. We also detected a significant time effect in the ADL subscale (P < 0.001, 95% CI=[−6.997, −2.570]). The scores increased in both groups from week_0_ to week_7_.

### Correlation of increments in ΔHbO₂ and FAAM scores

**Fig 6:**
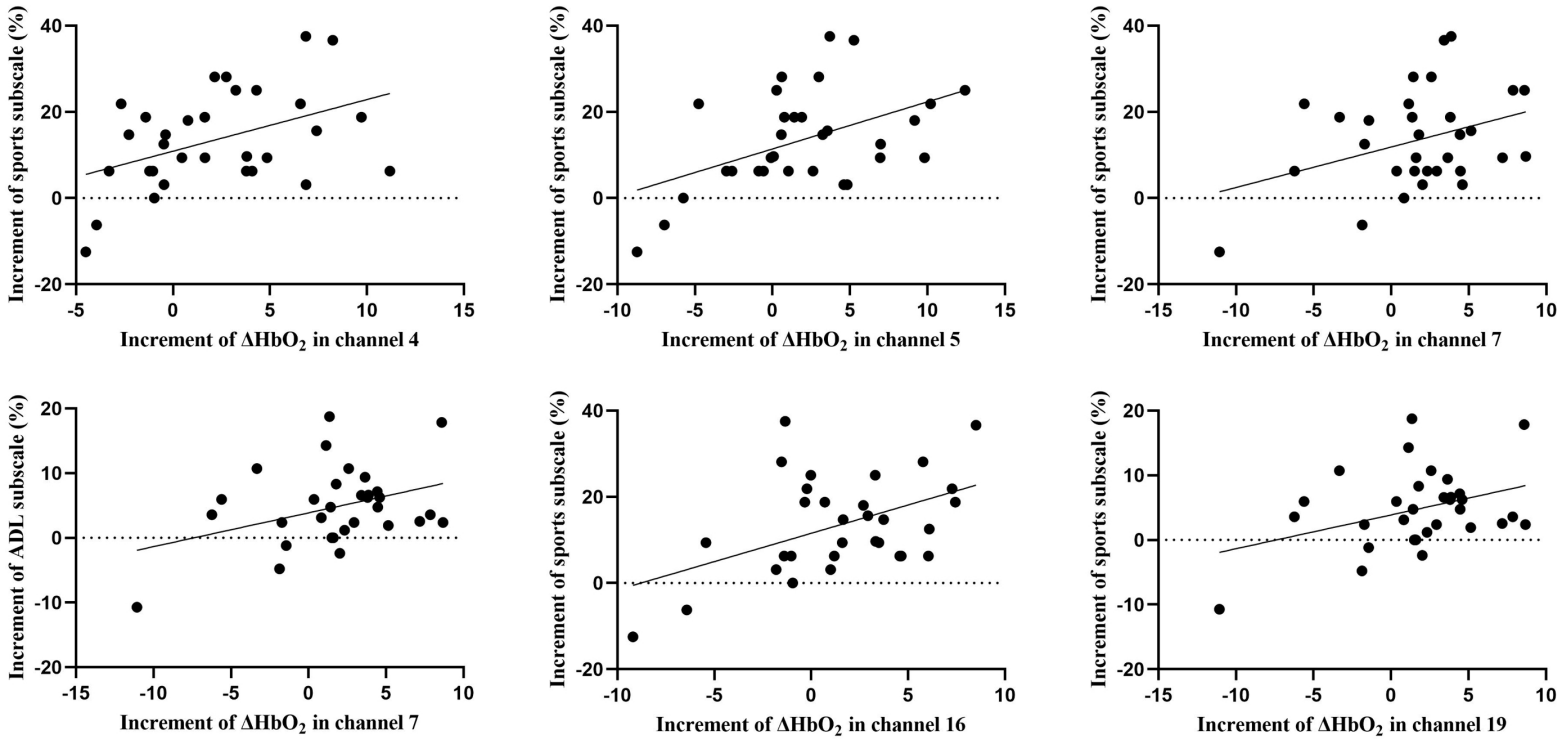
The ΔHbO_2_ increments of fNIRS channels that are significantly correlated with the increments in FAAM scores. ADL, activities of daily living; FAAM, Foot and Ankle Ability Measure.

The Shapiro-Wilk test showed that the increments of ΔHbO₂ in channel 14, channel 15 and channel 18 were not normally distributed (P < 0.05). Fig 6 shows that the increments of ΔHbO_2_ in channels 4, 5, 16, and 19 was moderately positively correlated with the increment of scores of the FAAM Sports subscale (P = 0.015, r = 0.434; P = 0.006, r = 0.480; P = 0.008, r = 0.470; P = 0.003, r = 0.513). The increment of ΔHbO_2_ in channel 7 was weakly positively correlated with the increment of scores of the FAAM Sports (P = 0.049, r = 0.356) and the ADL subscale (P = 0.038, r = 0.374).

## Discussion

Our study explored the effects of active or sham HD-tDCS with Bosu-ball training on cortical activation and ankle-foot function among individuals with CAI. Hypotheses # 1, 2 and 3 were supported, both interventions increased the cortical activation of the affected and non-affected side PMC & SMA, M1, S1 and SAC during single-leg stance, and increased the scores of the FAAM Sports and ADL subscales, while active HD-tDCS with Bosu-ball training showed a better effect in increasing the cortical activation of the affected and non-affected side PMC & SMA and the affected side M1, and increased the scores of the FAAM Sports subscale compared to sham HD-tDCS with Bosu-ball training among individuals with CAI. The increments in ΔHbO_2_ of the affected side PMC & SMA and S1, the non-affected side SAC and the affected and non-affected side M1 were positively correlated with the increments in scores of the FAAM Sports and ADL subscales.

A total of eleven longitudinal studies have explored the effectiveness of active tDCS on individuals with CAI [9,17–26], and three studies included cortical activity characteristics among this population [9,23,24]. The present study and Bruce et al. both observed significant active tDCS-induced neuroplastic changes. The present study found that active HD-tDCS with Bosu-ball training increased ΔHbO₂ in both the affected side and non-affected side PMC & SMA and the affected side M1 more significantly than sham HD-tDCS with Bosu-ball training, while Bruce et al. reported that active tDCS with strength training reduced resting motor threshold (RMT) in the peroneus longus, and reduced the I_50_ (intensity at peak slope) in both the peroneus longus and tibialis anterior more significantly than sham tDCS with strength training [9]. On the contrary, Beyraghi et al. and Needle et al. did not observe significant active tDCS-induced neuroplastic changes. Beyraghi et al. found no significant group-by-time interactions but time effects for late contingent negative variation (CNV) amplitude (C3, C4 and Cz), peak CNV amplitude (C3) within the groups that received active tDCS with balance training and sham tDCS combined with balance training [24]; Needle et al. did not report any significant changes in RMT, MEPₘₐₓ:Mₘₐₓ ratio, or H-reflex (Hₘₐₓ:Mₘₐₓ) within the groups that received active tDCS on M1, DLPFC and sham tDCS with motor planning training [23].

All four studies adopted active tDCS as the non-invasive brain stimulation, targeting sensorimotor-related brain regions (e.g., M1 [9,23], SMA [24], DLPFC [23], etc.) to explore cortical activity changes among individuals with CAI. Quantifiable neurophysiological or neuroimaging techniques were used to measure cortical activity (transcranial magnetic stimulation (TMS) [9,23], EEG [24] or fNIRS), and the intervention duration (4–6 weeks, 2–3 sessions/week [9,23,24]) was comparable. However, differences exist in four aspects. First is the tDCS configuration and targeting area. The present study used HD-tDCS with a 4 × 1 ring electrodes arrangement, placing the anode at Cz. In contrast, Bruce et al. and Needle et al. applied conventional tDCS (1.5 mA and 2 mA, respectively), targeting the affected-side M1 [9] and both the affected side M1 and the DLPFC [23]. And Beyraghi et al. applied conventional tDCS (1.5 mA) over the SMA (ahead of Cz) [24]. Second is the measurement tools. The present study employed fNIRS to assess task-related cortical activation, quantified as ΔHbO₂ in both the affected side and non-affected side PMC & SMA, M1, S1, and SAC. Bruce et al. and Needle et al. used TMS to measure resting-state corticospinal excitability, including RMT, I₅₀, and cortical silent period (CSP), etc. for peroneus longus (PL), and tibialis anterior (TA) [9], and RMT, and MEPₘₐₓ:Mₘₐₓ in PL, TA and Soleus [23]. Beyraghi et al. utilized EEG to capture preparatory brain activity, specifically late CNV amplitude, peak amplitude, and peak time at C3/Cz/C4 electrodes [24]. Third is the testing scenarios. fNIRS measurements were conducted during single-leg standing in the present study, which matches the balance training program. Bruce et al. and Needle et al. measured corticospinal excitability in a resting state (independent of training programs) [9,23], while Beyraghi et al. measured neural activity during the preparatory phase before gait initiation (indirectly related to training programs) [24]. Fourth is the physical training methods paired with tDCS. The present study implemented a 6-week progressive Bosu-ball balance training program synchronously with HD-tDCS, with multi-directional perturbations and requiring for continuous postural adjustments. Bruce et al. adopted 4-week eccentric strength training of the peroneus longus synchronously with tDCS [9], while Beyraghi et al. used a comprehensive balance training program (including single-leg stance, hopping, etc.) after tDCS intervention [24], and Needle et al. applied a motor planning training program (including obstacle walking, dual-task balance, etc.) synchronously with tDCS [23].

Inconsistent findings exist in cortical activity outcomes among these studies. Three factors may explain these discrepancies. First is the tDCS targeting specificity. HD-tDCS applied in the present study can achieve focal electric field distribution, concentrating stimulation on both the affected side and non-affected primary sensorimotor cortex (PSC, including S1 and M1) [17]. In contrast, conventional tDCS applied in the other two studies with no significant active tDCS-induced neuroplastic changes had relatively large electrode areas and diffused electric fields [27]. Beyraghi et al. targeted the SMA [24], and Needle et al. targeted the affected side M1 and DLPFC [23]. Second is the differences in testing scenarios. Task-specific measurements (single-leg stance) in the present study matches the Bosu ball training programs. Single-leg stance is an indispensable physical activity in daily life [48], as well as a feasible, challenging balance task [49]. Since individuals with CAI exhibit balance and postural control deficits, which are associated with functional and structural changes in cortical regions related to sensorimotor function [7], a challenging balance task like single-leg stance may elicit distinct and specific cortical activity among this population. However, resting-state TMS measurements applied in Needle et al. reflected baseline corticospinal excitability [23], while pre-motor state EEG measurements in Beyraghi et al. captured pre-movement preparatory brain activity only [24]. Third is the physical training methods paired with tDCS. The present study implemented a Bosu-ball balance training program synchronously with HD-tDCS, with multi-directional perturbations to the ankle and center of gravity [26]. Bosu ball training can simulate the scenario when an ankle sprain occurs, and can be regarded as a kind of motor learning process for the trainees to learn how to counteract these perturbations [26]. Based on the synchronous application of Bosu-ball training, tDCS can exert its effect on promoting motor learning [50–52]. Beyraghi et al. adopted a comprehensive balance training program after tDCS intervention [24]. The timing of physical training administration in combined interventions (tDCS with physical training) may affect the efficacy of tDCS. Study showed that voluntary muscle contraction (VMC) performed after tDCS can reduce or even tend to reverse the effects of tDCS on motor cortical excitability [53]. Specifically, the excitability enhancement induced by anodal tDCS is attenuated, and the excitability reduction induced by cathodal tDCS is counteracted [53]. This suggests that physical activity after tDCS may affect its induction of neuroplastic changes. Needle et al.’s motor planning training program was conducted synchronously with tDCS (training initiated after 2 min tDCS). The training program paired with M1-targeted or DLPFC-targeted active tDCS involved cognitive function modulation, and DLPFC-targeted active tDCS was mainly focused on motor planning (cognitive function), while indicators measured in this study (RMT, MEP, H-reflex) were corticospinal function (sensorimotor cortex/M1). This “mismatch” may lead to non-significant differences in neural outcomes.

Among the eleven longitudinal studies that explored the effectiveness of active tDCS on individuals with CAI [9,17–26], two studies included FAAM scores among this population [9,23]. Only the present study observed significant active tDCS-induced changes in FAAM subscale scores. The present study found that active HD-tDCS with Bosu-ball training increased the scores of FAAM Sports subscale more significantly than sham HD-tDCS with Bosu-ball training, while Bruce et al. and Needle et al. did not observe significant active tDCS-induced changes in FAAM subscale scores. Needle et al. found no significant group-by-time interactions but time effects in scores of FAAM Sports or ADL subscales within the groups that received active tDCS on M1, DLPFC and sham tDCS with motor planning training [23], while Bruce et al. did not report any significant changes in scores of FAAM Sports or ADL subscales within the groups that received active tDCS or sham tDCS with strength training [9].

Inconsistent findings exist in scores of FAAM subscales among studies that explored the effectiveness of tDCS on foot and ankle function among individuals with CAI. Two factors may explain these discrepancies. First is the tDCS targeting specificity as discussed above. Specifically, the DLPFC-targeted tDCS applied in Needle et al.’ s study mainly focused on motor planning (cognitive function) [23], while FAAM mainly involves foot and ankle sports and ADL function. Second is the FAAM baseline levels. Bruce et al. exhibited relatively high baseline scores of FAAM subscales (ADL subscales: 93.69 ± 5.33% in the aTDCS group, 92.74 ± 7.26% in the sham group; Sport subscales: 84.37 ± 12.88% in the aTDCS group, 79.37 ± 18.05% in the sham group), approaching the maximum possible score (100%) [9], which may lead to a ceiling effect that limited measurable improvements. Needle et al. exhibited relatively lower baseline scores of FAAM subscales (ADL subscales: 86.9 ± 14.2% in the motor group; 90.7 ± 9.5% in the frontal group, 85.6 ± 11.9% in the sham group; Sport subscales: 75.3 ± 18.6% in the motor group; 75.5 ± 20.8% in the frontal group, 68.7 ± 17.4% in the sham group) [23], which may allow sufficient space for measurable improvement.

Our results showed that Bosu-ball training increased the cortical activation of the affected side and non-affected side PMC & SMA, M1, S1, and SAC during single-leg stance among individuals with CAI. The finding is partially supported by a previous study, which indicated that a 6-week rehabilitation protocol increased the activation of affected side S1 during single-leg stance among individuals with CAI [54]. Our findings can be attributed to neuroplastic changes induced by Bosu-ball training through increasing sensory input to the sensorimotor area. Bosu-ball training can provide the affected ankle with multi-directional perturbations through the unstable hemispherical surface of Bosu-ball, which continuously stimulates the proprioceptors within the joint capsule and ligaments [26]. For the affected side S1 and SAC, the stimulation can promote the restoration of impaired proprioception of the affected ankle, and lead to an increased sensory input to the somatosensory cortex contralateral to the affected limb (i.e., the “affected side” S1 and SAC in the present study) [32]. This is because proprioceptive information originating from proprioceptors (e.g., mechanoreceptors in joint capsules and tendons) on one side is transmitted via the dorsal column-medial lemniscus pathway, relayed through the ventral posterolateral nucleus of the thalamus, and ultimately projected to the contralateral S1 [55]. S1 is mainly responsible for receiving and processing somatosensory information [56], while also participating in the formation of descending motor control commands [57]. SAC is mainly involved in the integration of inputs from other sensorimotor areas, contributing to sensorimotor integration [58]. However, SAC does not directly receive signals from peripheral receptors [59]. The core components of SAC-the lateral parietal cortex and posterior parietal cortex-primarily receive sensory input from the ipsilateral anterior parietal cortex, which includes the S1 [59]. Therefore, the reception and processing of sensory information by the SAC is also indirectly originate from the contralateral affected limb. During Bosu-ball training, when the affected lower limb stands on the unstable spherical surface of the Bosu-ball, the non-affected lower limb has to maintain specific joint postures (e.g., during single-leg stance, single-leg stance with knee bend and single-leg squat movements) or continuously adjust joint positions and movement directions or speeds (e.g., during single-leg stance with leg swing forward-backward and medially-laterally, swallow balance, etc.) to maintain balance. For the non-affected side S1 and SAC, frequent joint movements of the non-affected lower limb can also stimulate proprioceptors within the joint capsule and ligaments, increasing the proprioceptive input to the somatosensory cortex contralateral to the non-affected limb (i.e., the “non-affected side” S1 and SAC in the present study). Thus, both the affected and non-affected side S1 and SAC need to receive, process and integrate increased sensory information, and the activation of these cortical areas can increase during single-leg stance among individuals with CAI.

The increased activation of the affected side and non-affected side PMC & SMA and M1 can be attributed to increased input from the somatosensory cortex (S1 and SAC), which in turn leads to a greater activity in the formulation of motor commands and motor programs. For the affected side PMC & SMA and M1, after integrating and processing increased proprioceptive signals from the affected ankle, S1 and SAC contralateral to the affected limb (i.e., the “affected side” S1 and SAC in the present study) can transmit the enhanced sensory input to the ipsilateral motor cortex (i.e., the “affected side” PMC & SMA and M1 in the present study), thereby promoting the activity in motor command formulation and motor program planning [59]. Specifically, M1 generates descending motor commands to regulate postural control [57], while PMC & SMA are involved in motor learning and planning, and the formation of new motor programs [60]. There exists interhemispheric functional connections within the motor cortex (PMC & SMA, and M1) [61], which facilitate the completion of tasks even when one limb is primarily responsible for the execution of tasks [61,62]. Therefore, for the non-affected side PMC & SMA and M1, once the affected side motor cortex (PMC & SMA and M1) receives the enhanced sensory input from the ipsilateral somatosensory cortex (i.e., the “affected side” S1 and SAC in the present study), it can then transmit signals to the non-affected side motor cortex (PMC & SMA and M1) through the interhemispheric pathways. This signal transmission can also lead to an increased activity in motor command formulation and motor program planning of the non-affected side motor cortex (PMC & SMA and M1), enabling the non-affected limb to perform fine adjustments (e.g., during single-leg stance) and thereby promoting the postural control of both the affected and non-affected side limbs [7]. Such interhemispheric coordination ultimately facilitates the completion of the single-leg stance task, even though the affected limb is primarily responsible for weight-bearing and task execution. Thus, the activity of motor command formulation and motor program planning increases among both the affected and non-affected side PMC & SMA and M1, and the activation of these cortical areas can increase during single-leg stance among individuals with CAI.

Our results also showed that active HD-tDCS with Bosu-ball training can further increase the cortical activation of the affected side and non-affected side PMC & SMA and the affected side M1 during single-leg stance among individuals with CAI. The finding is partially supported by a previous study, which indicated that the corticospinal excitability of M1 to the lower limb muscles improved more after receiving four-week active tDCS with muscle strength training compared to sham tDCS with muscle strength training among individuals with CAI [9]. Our finding can be attributed to the effects of synchronous HD-tDCS on the process of motor learning and learning-related cortical areas during Bosu-ball training. Motor learning involves acquiring new motor skills, accompanied by neuroplastic changes in the learning-related cortical areas, like PMC & SMA and M1 [63]. Among them, M1 plays a dominant role in motor execution, and is a key structure in motor learning that can be modulated by non-invasive brain stimulation like tDCS [52]. Bosu-ball training can be regarded as a continuous learning process in the present study, as it exposes individuals with CAI to multi-directional affected ankle joint and center-of-gravity perturbations during the performance of a series of training movements [26]. During the learning process, individuals with CAI must continuously learn how to maintain balance while counteracting perturbations primarily by the affected lower limb (since the affected ankle was on the unstable spherical surface of the Bosu-ball). Consequently, for the affected side PMC & SMA and M1, activities of these learning-related cortical areas may then increase to make the trainee acquire motor skills [51]. The application of synchronous HD-tDCS may further augment the activation of learning-related cortical areas during such learning process through inducing following neuroplastic changes. Firstly, tDCS can enhance the formation of synaptic connections and regulate its plasticity [50], which is the key neuromechanism of motor learning. Secondly, tDCS can increase the cortical excitability of learning-related regions during learning process [51], achieved by changing the resting membrane potential and reducing the depolarization threshold of the membrane of neurons [14], making the neurons susceptible to activate. Formation of new synaptic connections and increased neuronal excitability within the learning-related cortical areas indicate a tendency to produce greater neuronal activities to acquire motor skills during the motor learning process. Thirdly, the reinforcement of synchronous tDCS on motor learning and learning-related cortical areas can be retained. Since synchronous tDCS can promote the long-term potentiation of neurons within targeted cortical areas during learning process [51], thus enhancing off-line motor learning (motor skill retention and consolidation) [52]. For the non-affected side PMC & SMA, the enhanced activation is primarily induced by the increased interhemispheric input signals it receives, which are derived from the affected side motor cortex (PMC & SMA and M1) induced by the tDCS. Specifically, PMC & SMA can mediate coordinated movements of bilateral limbs [7], since the bilateral PMC & SMA are structurally connected via fiber tracts in the corpus callosum, forming bidirectional interhemispheric functional connections [64]. As discussed above, the enhancement of cortical excitability and promotion of synaptic connectivity in the affected side motor cortex (PMC & SMA and M1) induced by HD-tDCS suggest increased neuronal firing frequency and synaptic transmission efficacy within these areas [65]. This may further increase the signals transmitted from the affected side PMC & SMA to the non-affected side PMC & SMA for maintaining bilateral limb coordinated movements during single-leg stance on the affected limb. For the non-affected side PMC & SMA, these enhanced interhemispheric input signals might then strengthen activities related to motor learning and planning, and the formation of new motor programs [60], ultimately leading to an increased activation.

Our results showed that Bosu-ball training increased the scores of FAAM sports and ADL subscales. The finding is partially supported by previous studies, which indicated that 6-week sham HD-tDCS with Bosu-ball training decreased the injury potential during drop-landing [26] and side-cutting tests [20], and improved postural stability among individuals with CAI [19]. Our finding can be attributed to the training effects of the unstable hemispherical surface of Bosu-ball. Bosu-ball delivers continuous multi-directional perturbations to the ankle joint, which in turn stretches the periarticular soft tissues, stimulates the mechanoreceptors within these tissues, and then induces reflexive muscle contraction in stabilizing the ankle joint, ultimately enhancing the strength of muscles around the ankle [31]. Besides, since the mechanoreceptors are under substantial stimulation induced by the Bosu-ball, the γ-motor neurons can be activated, and the sensory inputs increase, which in turn contributes to the restoration of impaired proprioception [32]. Another study showed that balance training on an unstable surface yields more significant improvement in balance and postural control than balance training on stable surface among individuals with CAI [40]. This is because balance training on an unstable surface can enhance body’s balance response by continuously disrupting balance achieved by the multi-directional perturbations it provides to the ankle joint [41].

Our results also showed that active HD-tDCS with Bosu-ball training can further increase the scores of FAAM sports subscale. The finding is partially supported by previous studies, which indicated that 6-week active HD-tDCS with Bosu-ball training decreased the injury potential during drop-landing [26] and side-cutting tests [20], and improved postural stability more significantly than sham HD-tDCS with Bosu-ball training among individuals with CAI [19]. Another study indicated that active tDCS with short foot exercise (SFE) can improve proprioception and dynamic postural control more significantly than sham tDCS with SFE among individuals with CAI [17]. Our finding can be attributed to the enhancing effects of synchronous HD-tDCS based on Bosu-ball training on motor learning and motor function. As discussed above, synchronous tDCS can facilitate motor learning, which may in turn augment the improvement of Bosu-ball training on ankle-foot function. Besides, tDCS can increase the excitability of descending corticospinal pathways projecting from M1 to lower limb muscles, which can in turn enhance muscle activation, thus improving motor function [26].

Our results showed that the increments in ΔHbO_2_ of the affected side PMC & SMA and S1, non-affected side SAC and the affected and non-affected side M1 are positively correlated with the increments in scores of the FAAM Sports and ADL subscales. The finding is partially supported by previous studies, which indicated that increased cortical activation of PMC & SMA, M1 and S1 correlated with improved motor performance during single-leg stance [54,66]. Our finding can be attributed to the fact that increased cortical activity levels enable individuals with CAI to mobilize sufficient neural resource for maintaining motor performance. Because increased activation of cortical areas associated with sensorimotor function indicates improved ability to integrate and process sensory information [54], as well as to control the activation of lower limb muscles [67]. These enhancements may contribute to the discrimination and adjustment of foot position, and sufficient activation of evertors to resist excessive ankle inversion during high injury risk scenarios such as drop-landing or side-cutting among individuals with CAI, ultimately preventing the occurrence of recurrent sprains.

This study has limitations. Firstly, no follow-up assessments were conducted post-intervention, so long-term effects of the intervention on both cortical activation and ankle-foot function among individuals with CAI remain unclear. Secondly, regarding participant blinding, the sham HD-tDCS settings followed commonly used guidelines in tDCS studies – simulating initial sensations of active HD-tDCS (e.g., tingling, itching), but there still may exist residual biological effects beyond the stimulation phase. Thirdly, the single-center design and recruitment from a highly selective student population limit the external validity and generalizability in a broader and more diverse population.

## Conclusions

Bosu-ball training increases cortical activation and improves ankle-foot function among individuals with CAI, and the increment of cortical activation is positively correlated with the improvement of ankle-foot function; Active HD-tDCS with Bosu-ball training increases cortical activation of both the affected and non-affected side PMC & SMA and affected side M1, and improves ankle-foot motor function more significantly than sham HD-tDCS with Bosu-ball training. These findings can be helpful in formulating rehabilitation programs targeting impaired cortical activation among individuals with musculoskeletal injuries in the future.

## Supporting information

S1 Table MNI coordinates of fNIRS channels and corresponding brain areas. MNI, Montreal Neurological Institute; fNIRS, functional near-infrared spectroscopy; PMC & SMA, premotor cortex and supplementary motor area; M1, primary motor cortex; S1, primary somatosensory cortex; SAC, somatosensory association cortex.
(DOCX)

S2 Table Adverse Event Record Form. (DOCX)

S3 Table Demographic data (M ± SD). FAAM, Foot and Ankle Ability Measure; M, mean values; SD, standard deviation; CAIT, Cumberland Ankle Instability Tool; tDCS, transcranial direct current stimulation.
(DOCX)

S4 Table The scores of the FAAM Sports and ADL subscales in two groups before and after the intervention (M ± SD). * Indicates significant statistical differences (P < 0.05); ^a^ Indicates significant statistical differences between week_0_ and week_7_ (P < 0.05). FAAM, Foot and Ankle Ability Measure; ADL, activities of daily living; M, mean values; SD, standard deviation; tDCS, transcranial direct current stimulation; 95% CI, 95% confidence interval.
(DOCX)

S1 Checklist CONSORT checklist. (DOCX)

S1 File Inclusivity in global research questionnaire. (DOCX)

S1 Data Brodmann Area Overlap Percentages for 20 Channels and 16 Optodes. (TXT)

S1 Text The trial protocol approved by the ethics committee. (PDF)

S2 Text The trial protocol approved by the ethics committee_translated. (PDF)
