# Supplementary material for: Transcranial direct current stimulation with Bosu-ball training increases cortical activation and improves ankle-foot function among individuals with chronic ankle instability: A randomized controlled trial
Source: PLoS One. 2026 Feb 27;21(2):e0342751. doi: 10.1371/journal.pone.0342751 (PMC12948058; doi:10.1371/journal.pone.0342751)
Supplement: S1 Table — MNI, Montreal Neurological Institute; fNIRS, functional near-infrared spectroscopy; PMC & SMA, premotor cortex and supplementary motor area; M1, primary motor cortex; S1, primary somatosensory cortex; SAC, somatosensory association cortex. (DOCX) [file pone.0342751.s001.docx]

**Supporting information**

**S1 Table.** **MNI coordinates of fNIRS channels and corresponding brain areas.**

| Channels | MNI coordinates | | | Brain areas | Coverage percentage |
| --- | --- | --- | --- | --- | --- |
|  | x | y | z |  |  |
| 1 | -23 | 6 | 71 | Left PMC & SMA | 92% |
| 2 | -33 | -5 | 68 |  | 100% |
| 3 | -14 | -8 | 77 |  | 100% |
| 4 | -23 | -19 | 76 |  | 68% |
| 5 | -37 | -28 | 71 | Left M1 | 71% |
| 6 | -14 | -30 | 79 |  | 91% |
| 7 | -24 | -71 | 75 | Left S1 | 62% |
| 8 | -35 | -55 | 69 | Left SAC | 77% |
| 9 | -16 | -57 | 74 |  | 100% |
| 10 | -24 | -68 | 68 |  | 100% |
| 11 | 18 | 9 | 72 | Right PMC & SMA | 94% |
| 12 | 8 | -5 | 75 |  | 100% |
| 13 | 26 | -3 | 72 |  | 100% |
| 14 | 15 | -16 | 78 |  | 75% |
| 15 | 8 | -31 | 80 | Right M1 | 96% |
| 16 | 26 | -27 | 76 |  | 73% |
| 17 | 16 | -42 | 80 | Right S1 | 51% |
| 18 | 9 | -57 | 74 | Right SAC | 100% |
| 19 | 26 | -56 | 74 |  | 100% |
| 20 | 18 | -68 | 69 |  | 100% |

MNI, Montreal Neurological Institute; fNIRS, functional near-infrared spectroscopy; PMC & SMA, premotor cortex and supplementary motor area; M1, primary motor cortex; S1, primary somatosensory cortex; SAC, somatosensory association cortex.
