# Supplementary material for: Transcranial direct current stimulation with Bosu-ball training increases cortical activation and improves ankle-foot function among individuals with chronic ankle instability: A randomized controlled trial
Source: PLoS One. 2026 Feb 27;21(2):e0342751. doi: 10.1371/journal.pone.0342751 (PMC12948058; doi:10.1371/journal.pone.0342751)
Supplement: S2 Table — (DOCX) [file pone.0342751.s002.docx]

| **Module** | **Specific Items** | **Instructions (Mandatory items marked with *)** |
| --- | --- | --- |
| 1. Participant Basic Information | *Participant Anonymous ID: __________  *Age: ______ years  *Gender: □ Male □ Female  *Intervention Group: □ tDCS+Bosu Group □ Bosu Group  *Contact Information (Optional): __________ | ID should follow the study numbering rule (e.g., T01/B02). Contact information is for follow-up and can be anonymized. |
| 2. Basic Information of Adverse Event | *Time of Event Onset: ______ Year ____ Month ____ Day ____ Hour ____ Minute  *Event Type: □ tDCS-related □ Bosu-ball training-related □ Other (specify): __________  *Specific Manifestations (Multiple choices allowed): □ Scalp tingling □ Scalp itching □ Muscle soreness □ Ankle discomfort □ Dizziness □ Nausea □ Minor bump □ Other (specify): __________  *Severity: □ Mild (no treatment needed, no impact on intervention) □ Moderate (intervention adjustment required, no medical treatment) □ Severe (medical treatment needed, study interruption) | Specify the location for manifestations (e.g., “right calf muscle soreness”, “left scalp tingling”). Check the severity based on the definition. |
| 3. Event Correlation Information | *Correlated Intervention: □ tDCS Session ___ □ Bosu-ball Training Session ___ □ Assessment Process (specify): __________  *Duration of Event: ______ hours/days  *Intervention Interruption: □ Yes (Duration of interruption: ______) □ No | Fill in the specific session number for correlated intervention (e.g., “3rd tDCS session”). Duration refers to the time from onset to remission. |
| 4. Management and Follow-up | *Management Measures (Multiple choices allowed): □ No special treatment □ Pause current intervention □ Adjust tDCS current/electrode position □ Local cold/hot compress □ Other (specify): __________  *Follow-up Time 1: ______ Year ____ Month ____ Day (1 day after event)  Follow-up Outcome 1: □ Fully recovered □ Improved □ No change □ Worsened  *Follow-up Time 2: ______ Year ____ Month ____ Day (3 days after event)  Follow-up Outcome 2: □ Fully recovered □ Improved □ No change □ Worsened  *Follow-up Time 3: ______ Year ____ Month ____ Day (1 week after event)  Follow-up Outcome 3: □ Fully recovered □ Improved □ No change □ Sequelae (specify): __________ | Fill in actual management measures; write “None” if no follow-up is conducted. Clearly state symptom changes for follow-up outcomes. |
| 5. Documentation and Review | *Recorder’s Signature: __________  *Documentation Date: ______ Year ____ Month ____ Day  *Reviewer’s (Principal Investigator) Signature: __________  *Remarks: __________ | Remarks can include supplementary information (e.g., “Symptom disappeared spontaneously after the 2nd intervention”). |
