# Supplementary material for: Transcranial direct current stimulation with Bosu-ball training increases cortical activation and improves ankle-foot function among individuals with chronic ankle instability: A randomized controlled trial
Source: PLoS One. 2026 Feb 27;21(2):e0342751. doi: 10.1371/journal.pone.0342751 (PMC12948058; doi:10.1371/journal.pone.0342751)
Supplement: S3 Table — FAAM, Foot and Ankle Ability Measure; M, mean values; SD, standard deviation; CAIT, Cumberland Ankle Instability Tool; tDCS, transcranial direct current stimulation. (DOCX) [file pone.0342751.s003.docx]

**Supporting information**

**S3 Table. Demographic data (M±SD).**

| FAAM | Bosu  (n=14) | tDCS+Bosu  (n=17) | t | P |
| --- | --- | --- | --- | --- |
| Age (years) | 21.21±1.72 | 20.47±0.94 | -1.450 | 0.163 |
| Height (cm) | 173.17±11.88 | 175.33±8.25 | 0.596 | 0.556 |
| Body mass (kg) | 68.91±11.61 | 71.14±8.35 | 0.624 | 0.538 |
| CAIT Scores | 16.00±3.94 | 15.59±5.28 | -0.241 | 0.811 |

FAAM, Foot and Ankle Ability Measure; M, mean values; SD, standard deviation; CAIT, Cumberland Ankle Instability Tool; tDCS, transcranial direct current stimulation.
