# Supplementary material for: Transcranial direct current stimulation with Bosu-ball training increases cortical activation and improves ankle-foot function among individuals with chronic ankle instability: A randomized controlled trial
Source: PLoS One. 2026 Feb 27;21(2):e0342751. doi: 10.1371/journal.pone.0342751 (PMC12948058; doi:10.1371/journal.pone.0342751)
Supplement: S4 Table — * Indicates significant statistical differences (P < 0.05); a Indicates significant statistical differences between week0 and week7 (P < 0.05). FAAM, Foot and Ankle Ability Measure; ADL, activities of daily living; M, mean values; SD, standard deviation; tDCS, transcranial direct current stimulation; 95% CI, 95% confidence interval. (DOCX) [file pone.0342751.s004.docx]

**Supporting information**

**S4 Table. The scores of the FAAM Sports and ADL subscales in two groups before and after the intervention (M±SD).**

| FAAM | Week | Bosu  (n=14) | tDCS+Bosu  (n=17) | Interaction | | | Time | | | Group | | |
| --- | --- | --- | --- | --- | --- | --- | --- | --- | --- | --- | --- | --- |
|  |  |  |  | F | P | η^2^_p_ | F | P | 95% CI | F | P | 95% CI |
| Sports-  subscales | 0 | 78.57±10.32 | 74.14±12.67 | 4.326 | 0.046* | 0.130 | 45.147 | <0.001 | -17.688, -9.325 | 0.016 | 0.900 | -5.787, 6.546 |
|  | 7 | 87.63±8.75^a^ | 91.31±6.75^a^ |  |  |  |  |  |  |  |  |  |
| ADL-  subscales | 0 | 91.03±6.48 | 89.49±7.42 | 2.052 | 0.163 | 0.066 | 18.756 | <0.001* | -6.997, -2.570 | <0.001 | 0.997 | -3.493, 3.509 |
|  | 7 | 94.13±6.82 | 95.65±5.00 |  |  |  |  |  |  |  |  |  |

* Indicates significant statistical differences (P<0.05); ^a^ Indicates significant statistical differences between week_0_ and week_7_ (P<0.05).

FAAM, Foot and Ankle Ability Measure; ADL, activities of daily living; M, mean values; SD, standard deviation; tDCS, transcranial direct current stimulation; 95% CI, 95% confidence interval.
