# Supplementary material for: Transcranial direct current stimulation with Bosu-ball training increases cortical activation and improves ankle-foot function among individuals with chronic ankle instability: A randomized controlled trial
Source: PLoS One. 2026 Feb 27;21(2):e0342751. doi: 10.1371/journal.pone.0342751 (PMC12948058; doi:10.1371/journal.pone.0342751)
Supplement: S1 Text — (PDF) [file pone.0342751.s008.pdf]

山东体育学院关于涉及人的医学研究项目伦理审查

申　请　书

山东体育学院运动科学伦理委员会：

兹将拟申报项目 波速球训练及同步经颅直流电刺激对慢性踝关节不稳者皮层激活的影响 提交贵委员会，请予以伦理审查。

第一部分　项目基本情况

1、研究项目名称： 波速球训练及同步经颅直流电刺激对慢性踝关节不稳患者皮层激活的影响

2、项目负责人： 罗心

主要参与人：高贺，黄雪可，葛玉斌，沈培鑫

联系人： 宋祺鹏 办公电话： ________ 手机： 15508619996 电子邮箱： songqipeng@sdpei.edu.cn

3、研究场所： 山东体育学院生物力学实验室

4、研究起止时间： 2024年8月——2024年11月

5、项目资金来源： 山东省教育厅“生物力学工程”国际合作联合实验室（山东体育学院--拉夫堡大学）

第二部分　研究计划方案

6、科学依据和背景（包括相关研究结果与动物试验结果）：

踝关节扭伤是体育运动中最为常见的损伤，约占所有运动损伤的10%~30%，其中外侧踝关节扭伤(lateral ankle sprains, LAS)占比达 80%~90%。急性踝关节扭伤后，高达约 70%的患者会在短时间内发展成为CAI。CAI常表现为踝关节处的疼痛、肿胀、不稳定感、踝关节失控(give way)及反复性扭伤，严重影响 CAI 者的身体功能及日常生活[5]。在美国，每年约发生200万例踝关节扭伤，年度医疗支出高达约200亿美元，为社会带来严重经济负担。一般意义上CAI被认为是一种伴有韧带功能缺陷的肌肉骨骼损伤，常规的干预方式如力量训练、关节松动术、平衡训练等，均以CAI者的局部症状以及身体功能障碍为治疗靶点，可在一定程度上缓解症状并改善CAI者的身体功能，但是由CAI引起的残疾率及再扭伤风险仍然居高不下，其原因可能是在于忽略了CAI者损伤后中枢神经系统出现的神经可塑性变化。最近，许多研究者提出CAI可被视为是一种伴有中枢神经系统内，尤其是大脑皮层，出现不良适应性神经可塑性变化的神经生理学障碍，这种可塑性变化会影响机体的感觉运动功能，可能是

诱导身体功能障碍和出现反复性扭伤的关键因素。经颅直流电刺激(transcranial direct current stimulation, tDCS)有望成为直接作用于肌肉骨骼损伤后大脑皮层不良适应性神经可塑性变化的有效干预措施。tDCS 通常作为一种辅助疗法与运动干预相结合以促进运动中技能习得的过程，进而改善身体功能，所以需考虑与之搭配的运动任务。波速球训练可以在一定程度上模拟踝关节扭伤，使训练者在训练过程中学习如何应对踝关节的扰动，而同步应用的 tDCS 可以促进这一习得过程，改善机体的运动功能。此外，将波速球训练这一类运动干预与 tDCS 相结合的联合干预方式能够较单一运动干预更好地促进适应性神经可塑性变化、调节大脑皮层活动。因此，本研究拟应用经颅直流电刺激联合波速球训练的干预方式，对比单一波速球训练的干预方式，探究基于波速球训练的同步经颅直流电刺激 CAI 人群皮层激活的影响。本研究从损伤后中枢神经系统的不良适应性神经可塑性变化出发，以大脑皮层为靶点应用不同途径的干预方式，以期为 CAI 人群的临床康复提供新思路，为制定中枢干预方案提供支持，实现该人群的全面康复。

7、研究目的：

本研究旨在通过对 CAI 人群进行为期 6 周的 tDCS 联合波速球训练和假 tDCS 联合波速球训练的干预方式，探讨基于波速球训练的同步 tDCS 对 CAI 人群皮层激活的影响。

8、受试者数目、招募方式及纳入/排除标准：

本研究通过张贴海报及线上招募形式在山东体育学院及山东建筑大学招募慢性踝关节不稳参与者共 40 人，每组各 20 人。根据国际足踝协会的建议和实验设计，将纳入和排除标准设为：

纳入标准：

(1) 一年前至少发生过一次严重踝关节扭伤，伴有疼痛、肿胀和其他炎性症状，超过一天不能正常参见正常日常活动。

(2) 18-24 岁，男女不限。

(3) 在过去的 6 个月内至少发生过两次踝关节“失控”。

(4) 日常生活中持续的踝关节不稳定感和功能障碍。

(5) 坎伯兰踝关节不稳评分(Cumberland Ankle Instability Tool, CAIT)得分 ＜ 24 分。

排除标准：

(1) 下肢发生过骨折或进行过手术。

(2) 三个月内发生过急性损伤如下肢扭伤。

(3) 双侧 CAI。

(4) 自我报告严重影响运动功能的神经系统疾病（癫痫等）。

9、研究方法(包括试验期限、进度，统计分析方法，以及对受试者的副作用如何处理)：

本试验所有实验对象均在山东体育学院生物力学实验室接受干预治疗。本研究采用随机、单盲、对照实验设计。拟将参与者通过随机生成的数字序列将其分为两组（每组 20 人）。一组参与者接受 tDCS 同步联合波速球训练（联合组），另一组参与者为避免安慰剂效应，接受假 tDCS 联合波速球训练（波速球组）。干预持续 6 周，共计 18 次干预（每周进行 3 次，每次 20 分钟，每次干预间隔 24 小时以上）。在干预前后分别进行 fNIRS 测试。本实验自 2023 年 10 月开始，至 2024 年 1 月结束，现已完成全部实验。使用 SPSS 21.0 软件进行统计分析。使用 Shapiro-Wilk 检验验证数据的正态性。数据若符合正态分布，则使用双因素重复测量方差分析来验证分组（联合组 vs 波速球组）与时间（干预前 vs 干预后）自变量的交互效应与主效应，若不符合正态分布，则采用 Scheirer-Ray-Hare 检验。如存在显著的交互效应，使用经 Bonferroni 调整的事后分析进行成对比较。显著性水平 α 为 0.05,偏 eta 平方（η^2_p）表示主效应及交互效应的效应大小。偏 eta 平方的阈值分别为:0.01-0.06，小；0.06-0.14，中等；＞0.14，大。使用 Cohen’s *d* 表示成对比较的效应大小。Cohen’s *d* 的阈值为:＜0.20，微弱；0.21-0.50，小；0.51-0.80，中等；＞0.81，大。若实验过程中对受试者产生的副作用则终止实验。

10、研究对象的选定

10.1 招募范围 健康者☐ 病人☑

10.2 是否对研究对象说明研究目的？ 是☑ 否☐

11、知情同意

11.1 将以何种形式获得研究对象的同意。 书面☑ 口头 ☐

11.1.1 不能以书面方式表达的原因：________________

11.1.2 由谁来向研究对象说明实验目的和要求：本实验主要参与人员

11.1.3 是否在必要时提供口头翻译？ 是☑ 否☐

11.2 如果研究对象（譬如儿童）不能表达意愿，将由谁来做决定？

研究对象的监护人或家人

12、保密

12.1 在研究期间及研究完成后，谁有权使用原始数据？

本研究的原始数据由本实验主要参与人员进行使用。

12.2 原始数据及资料如何保管？

原始数据将通过纸质，移动硬盘以及云端由本实验主要参与人员加密保存。

12.3 在论文或研究报告等研究成果中是否保证不公开个人姓名及足以让人识别出受试者身

份的信息？ 是☑ 否☐

13、风险评估

13.1 此研究是否可能导致对研究对象的精神伤害？ 是☐ 否☑

此研究是否可能导致对研究对象的躯体伤害？ 是☐ 否☑

此研究是否会增加研究对象的额外经济负担？ 是☐ 否☑

13.2 研究如果导致伤害，如何处理？

____

13.3 此研究是否涉及到个人隐私？ 是☐ 否☑

如果涉及到个人隐私，如何处理？

____

13.4 此研究是否涉及以下特殊研究对象？

子宫中胎儿 是☐ 否☑

无法成活的胎儿/流产的胎儿 是☐ 否☑

婴儿（0-1 岁） 是☐ 否☑

儿童（1-13 岁） 是☐ 否☑

少年（13-18 岁） 是☐ 否☑

孕妇/哺乳期妇女 是☐ 否☑

老人（60 岁以上） 是☐ 否☑

心智不全者 是☐ 否☑

如果涉及以上特殊研究对象，说明理由：____________________

如果涉及以上特殊研究对象，说明将如何采取特殊保护措施：____________

____

第三部分：其它

14、利益：

14.1 研究是否可能给社会带来益处？ 是☑ 否☐

14.2 研究是否给研究对象带来直接利益？ 是☐ 否☑

14.3 给研究对象支付的补偿性报酬，是否足以对之造成经济上的诱导？

是☐ 否☑

15、潜在的危害：

15.1 研究否存在潜在危害？ 是☐ 否☑

15.2 如果存在潜在危害，采取哪些预防措施？

15.3 是否给研究对象提供研究人员电话，以备咨询？ 是☑ 否☐

16、研究人员保证：

16.1 遵守世界医学协会（WMA）通过《赫尔辛基宣言》（2008 年修订版）所阐述的原则，世界卫生组织（WHO）国际医学科学理事会（CIOMS）合作的《涉及人的生物医学研究的国际伦理准则》（2002），以及联合国教科文组织（UNESCO）《世界人类基因组与人权宣言》（1997）中规定的伦理要求。

遵循国家食品药品监督管理局的《药物临床试验质量管理规范》（2003 年 9 月 1 日和卫生部的《涉及人的生物医学研究伦理审查办法（试行）》（2007 年 1 月 11 日）。

16.2 我们将尊重伦理委员会对本项目研究提出伦理建议，在研究工作进程中如发现涉及研究对象风险或不曾预料到的问题，随时与伦理委员会进行沟通。

16.3 我们将保守研究对象的个人隐私，做好保密工作，所有原始数据，相关文件材料，作机要档案保管，至少在研究结束后保管三年以上。

16.4 我们在研究过程中保存精确记录，以备检查总结。

项目负责人（签名）：

职务：学生

所在单位（盖章）：

时间：

所在单位负责人（签名）：
