# Supplementary material for: Transcranial direct current stimulation with Bosu-ball training increases cortical activation and improves ankle-foot function among individuals with chronic ankle instability: A randomized controlled trial
Source: PLoS One. 2026 Feb 27;21(2):e0342751. doi: 10.1371/journal.pone.0342751 (PMC12948058; doi:10.1371/journal.pone.0342751)
Supplement: S2 Text — (PDF) [file pone.0342751.s009.pdf]

Application for Ethical Review of Human-Involved Medical Research Projects of Shandong Sport University

To the Sports Science Ethics Committee of Shandong Sport University:

We hereby submit the proposed project titled "Effects of Bosu-ball Training Combined with Synchronous Transcranial Direct Current Stimulation on Cortical Activation in individuals with Chronic Ankle Instability" to your committee for ethical review.

Part 1: Basic Information of the Project

1. **Title of the Research Project:** Effects of Bosu-ball Training Combined with Synchronous Transcranial Direct Current Stimulation on Cortical Activation in individuals with Chronic Ankle Instability
2. **Project Leader:** Xin Luo
   Main Participants: He Gao, Xueke Huang, Yubin Ge, Peixin Shen
   Contact Person: Qipeng Song. **Office Phone:** None. **Mobile Phone:** 15508619996. **E-mail:** songqipeng@sdpei.edu.cn
3. **Research Venue:** Biomechanics Laboratory of Shandong Sport University
4. **Research Period:** August 2024-November 2024
5. **Source of Project Funding:** "Biomechanical Engineering" International Cooperation Joint Laboratory of Shandong Provincial Department of Education (Shandong Sport University-Loughborough University)

Part 2: Research Plan and Protocol

6. Scientific Basis and Background (Including Relevant Research Results and Animal Experiment Results)

Ankle sprains are the most common injuries in sports, accounting for approximately 10%-30 % of all sports injuries, among which lateral ankle sprains (LAS) account for 80%-90%. After acute ankle sprains, up to about 70% of patients will develop chronic ankle instability (CAI) within a short period of time. CAI is often characterized by pain, swelling, a sense of instability, ankle " giving way", and recurrent sprains, which seriously affect the physical function and daily life of individuals with CAI. In the United States, approximately 2 million ankle sprains occur each year, with annual medical expenditures reaching about 20 billion dollars, imposing a severe economic burden on society. Generally, CAI is regarded as a musculoskeletal injury accompanied by ligament defects. Conventional intervention methods such as strength training, joint mobilization, and balance training target the local symptoms and physical dysfunction of individuals with CAI. These methods can alleviate symptoms and improve the physical function of individuals with CAI to a certain extent, but the disability rate and risk of recurrent sprain caused by CAI remain high. The reason may be that the neuroplastic changes in the central nervous system (CNS) of individuals with CAI after injury are ignored. Recently, many researchers have proposed that CAI can be regarded as a neurophysiological disorder accompanied by Maladaptive neuroplastic changes in the CNS, especially in the cerebral cortex. Such neuroplastic changes would affect the sensorimotor function, which may be the key factor in inducing physical dysfunction and recurrent

sprains. Transcranial direct current stimulation (tDCS) is expected to be an effective intervention that directly targets the maladaptive neuroplastic changes in the cerebral cortex after musculoskeletal injuries. As an adjuvant therapy, tDCS is usually combined with physical training to promote the acquisition of motor skills during movement, thereby improving physical function. Therefore, the matching training methods need to be considered. Bosu-ball training can simulate ankle sprains to a certain extent, enabling trainers to learn how to respond to ankle disturbances during training, while synchronously applied tDCS can promote this learning process and improve the motor function. In addition, the combined intervention of Bosu-ball training (a type of physical training) and tDCS can further promote adaptive neuroplastic changes and regulate ccortical activity compared with single physical training. Therefore, this study intends to apply transcranial direct current stimulation combined with Bosu-ball training, compare it with single Bosu-ball training, to explore the effects of synchronous transcranial direct current stimulation based on Bosu-ball training on cortical activation in individuals with CAI. Starting from the maladaptive neuroplastic changes in the CNS after injury, this study targets the cerebral cortex with intervention methods through different approaches, aiming to provide new ideas for the clinical rehabilitation of CAI, support the formulation of new CNS intervention plans, and achieve comprehensive rehabilitation of this population.

7. Research Objective:

The objective of this study is to explore the effects of synchronous tDCS combined with Bosu-ball training on cortical activation in individuals with CAI, by implementing active tDCS and sham tDCS combined with Bosu ball training for 6 weeks.

8. Number of Participants, Recruitment Methods, and Inclusion/Exclusion Criteria:

A total of 40 participants with CA will be recruited from Shandong Sport University and Shandong Jianzhu University through poster advertisements and online recruitment. Participants will be divided into two groups with 20 in each group. In accordance with the recommendations of the International Ankle Consortium and the experimental design, the inclusion criteria are set as follows:

1. Participants experienced at least one severe ankle sprain one year prior, resulting in pain, swelling, and other inflammatory symptoms that hindered participation in daily activities for more than one day;
2. Particiapnts aged 18-24, regardless of gender;
3. Particiapnts have experienced at least two episodes of ankle "giving way" within the past six months;
4. Participants have a persistent feeling of ankle instability and functional impairment during daily activities;
5. Participants have a score<24 of the Cumberland Ankle Instability Tool (CAIT).

The exclusion criteria included:

1. Participants have a history of lower-extremity fracture or surgery within the past year;
2. Participants have experienced acute injuries such as lower-extremity sprains within the past three months;
3. Paritcipants have bilateral CAI;
4. Participants reported neurological disorders that affects motor function.

9. Research Methods (Including Trial Duration, Progress, Statistical Analysis Methods, and How to Handle Side Effects in Participants):

All participants in this experiment receive intervention at the Biomechanics Laboratory of Shandong Sport University. This study adopt a randomized, single-blinded, controlled experimental design. Participants will be divided into two groups (20 in each group) according to a randomly generated number sequence. tDCS+Bosu group receives synchronous active tDCS combined with Bosu ball training, and Bosu group receives sham tDCS combined with Bosu-ball training. The intervention last for 6 weeks, with a total of 18 sessions (3 sessions per week, 20 minutes per session, and an interval of more than 24 hours between each session. Functional near-infrared spectroscopy (fNIRS) test will be conducted before and after the intervention.

This experiment starts in August 2024 and ended in Novemember 2024. SPSS 21.0 software is used for statistical analysis, and the Shapiro-Wilk test is used to verify the normality of the data. If the data are normally distributed, a two-way analysis of variance (ANOVA) with repeated measures is used to verify the main effects of group (tDCS+Bosu group vs. Bosu group) and time (week$_0$ vs. week$_7$), and group-by-time interaction. If the data are not normally distributed, the Scheirer-Ray-Hare test would be adopted. If a significant interaction is detected, Bonferroni-adjusted post-hoc analysis would be used to conduct pairwise comparisons. Partial eta square (η^2_p) is used to represent the effect size of main effects and interactions. The thresholds for η^2_p are as follows: 0.01-0.06, small; 0.06-0.14, moderate; >0.14, large. Cohen' s d is used to represent the effect size of the post-hoc analysis. The thresholds for Cohen' s *d* are as follows: <0.20, trivial; 0.21-0.50, small; 0.51-0.80, medium; >0.81, large. The significance level α is set at 0.05. If adverse effects occur among participants during the experiment, the experiment would be terminated.

10. Selection of Research Participants

10.1 Recruitment scope

Healthy people; Patients (Selected)

10.2 Whether to explain the research purpose to the participants?

Yes (Selected) ; No

11. Informed Consent

11.1 Form of obtaining consent from participants:

Written (Selected); Oral

11.1.1 Reason for inability to express consent in written form:

N/A

11.1.2 Who will explain the research purpose and requirements to the participants?

Main personnel of this experiment

11.1.3 Whether to provide oral translation when necessary?

Yes (Selected) ; No

11.2 If the participants (e.g., Children) cannot express their wills, who will make the decision?

Guardians or family members of the participants.

12. Confidentiality

12.1 Who has the right to use the original data during and after the research?

The original data of this research will be used by the main personnel of this experiment.

12.2 How to store the original data and materials?

The original data will be encrypted and stored by the main personnel of this experiment in paper form, mobile hard drives and the cloud.

12.3 Whether to ensure that personal names and information sufficient to identify the participants are not disclosed in papers or research reports?

Yes (Selected) ; No

13. Risk Assessment

13.1 Is this research likely to cause psychological harm to the research participants?

Yes; No (Selected)

Is this research likely to cause physical harm to the research participants?

Yes; No (Selected)

Is this research impose additional economic burden on the research participants?

Yes; No (Selected)

13.2 If the research causes harm, how will it be handled?

N/A

13.3 Does this research involve personal privacy?

Yes; No (Selected)

If personal privacy is involved, how will it be handled?

N/A

13.4 Does this research involve the following special research subjects?

Fetus in the uterus

Yes; No (Selected)

Non-viable fetus/aborted fetus

Yes; No (Selected)

Infants (0-1 year old)

Yes; No (Selected)

Children (1-13 years old)

Yes; No (Selected)

Adolescents (13-18 years old)
Yes; No (Selected)
Pregnant women/breastfeeding women
Yes; No (Selected)
Elderly people (over 60 years old)
Yes; No (Selected)
People with mental incapacity
Yes; No (Selected)
If the above-mentioned special research participants are involved, please explain the reasons:
N/A
If the above-mentioned special research participants are involved, please explain the special protective measures to be taken:
N/A

Part 3: Other Matters

14. Benefits:
14.1 Is this research likely to bring benefits to society?
Yes (Selected) ; No
14.2 Does this research bring direct benefits to the research participants?
Yes; No (Selected)
14.3 Is the compensatory remuneration paid to research participants sufficient to create a financial inducement?
Yes; No (Selected)
15. Potential Harms:
15.1 Does the research have potential harms?
Yes; No (Selected)
15.2 If there are potential harms, what preventive measures will be taken?
N/A
15.3 Is the researcher's phone number provided to the research participants for consultation purposes?
Yes (Selected) ; No
16. Researchers' guarantee:
16.1 Abide by the principles set forth in the《Declaration of Helsinki (revised 2008)》adopted by the World Medical Association (WMA), the ethical requirements specified in the《International Ethical Guidelines for Biomedical Research Involving Human Subjects (2002)》collaborated on by the World Health Organization (WHO) and the Council for International Organizations of Medical Sciences (CIOMS), as well as those in the《Universal Declaration on the Human Genome and Human Rights (1997)》of the United Nations Educational, Scientific and Cultural Organization (UNESCO).
Abide by the《Good Clinical Practice》(September 1, 2003) issued by the State Food and Drug Administration, and the《Measures for the Ethical Review of Biomedical Research Involving Humans (Trial)》(January 11, 2007) by the Ministry of Health.
16.2 We respect the ethical suggestions put forward by the Ethics Committee regarding this research project. During the course of the research, if any risks to research participants or unforeseen issues are identified, we will communicate with the Ethics Committee timely.
16.3 We protect the personal privacy of research participants and ensure proper confidentiality. All original data and relevant documents will be kept as confidential archives and stored for at least three years after the completion of the research.
16.4 We will keep accurate records during the research process for inspection and summary purposes.

Project Leader (Signature):
Position: Student

Head of Affiliated Institution (Signature):

Affiliated Institution (Seal):
